# Associations between cardiovascular-kidney-metabolic syndrome staging and risks of all-cause and cardiovascular mortality: a systematic review and meta-analysis

**DOI:** 10.1016/j.ajpc.2026.101407

**Published:** 2026-01-02

**Authors:** Huimin Ding, Liqun Jiang, Yiqiu Zhao, Dongjun Lee, Buongo Chun

**Affiliations:** a Graduate School of Physical Education, Myongji University, Yongin 17058, , Republic of Korea; b Waseda University, 1-104 Totsukamachi, Shinjuku-ku, Tokyo, 169-8050, Japan

**Keywords:** Cardiovascular–kidney–metabolic syndrome, All-cause mortality, Cardiovascular mortality, Meta-analysis, Systematic Review

## Abstract

**Background:**

Cardiovascular, kidney, and metabolic disorders are closely interconnected and jointly increase atherosclerotic burden and mortality risk. In 2023, the American Heart Association introduced the cardiovascular–kidney–metabolic (CKM) syndrome as a staged framework to characterize this multisystem risk. However, evidence linking CKM stages to mortality has not yet been quantitatively synthesized.

**Methods:**

We conducted a PROSPERO-registered (CRD420251161180) systematic review and meta-analysis following MOOSE and PRISMA guidelines. Four databases (PUBMED,Web of science, Embase, Cochrane library)were searched through August 8, 2025. Cohort studies assessing CKM stages (0–4) and reporting all-cause, cardiovascular disease (CVD), coronary heart disease (CHD), or stroke mortality were synthesized using random-effects models, with subgroup, meta-regression, and sensitivity analyses.

**Results:**

9 cohort studies including 10,330,498 participants were analyzed. Mortality risk increased significantly with advancing CKM stages. Compared with Stage 0, Stage 4 was associated with markedly higher risks of all-cause mortality (HR = 3.82, 95% CI: 2.24–6.52), CVD mortality (HR = 6.38, 95% CI: 5.22–7.80), CHD mortality (HR = 9.19, 95% CI: 6.93–12.20), and stroke mortality (HR = 5.48, 95% CI: 4.28–7.02). Meta-regression identified age and educational attainment as significant effect modifiers and sources of heterogeneity. In sex-stratified analyses, all-cause mortality was consistently higher in women across CKM stages, whereas CVD mortality was higher in women at earlier stages but higher in men at later stages. Leave-one-out (LOO) sensitivity analyses and publication bias assessments supported the robustness of these findings.

**Conclusions:**

Advancing CKM stages are associated with progressively higher mortality risks, highlighting their value for early risk assessment and targeted prevention.

## 1 Background

Cardiovascular disease, chronic kidney disease, and metabolic abnormalities constitute the major global burden of chronic diseases [[1], [2], [3]]. These conditions interact through shared metabolic and inflammatory pathways, forming a complex pathological network that accelerates the development and progression of atherosclerosis and markedly increases the risk of all-cause and cardiovascular mortality (CVD mortality) [4,5]. To provide a unified framework for describing this cross-system pathophysiological process, the American Heart Association (AHA) proposed the concept of cardiovascular–kidney–metabolic (CKM) syndrome [6]. Specifically, CKM syndrome moves beyond individual disease risk factors by capturing the interdependent progression among the cardiovascular, kidney, and metabolic systems in a staged manner [4]. This enables an integrated assessment of the cumulative pathological burden that is often overlooked by traditional metabolic syndrome (MetS) or cardiovascular disease risk prediction models [7]. This framework highlights the cumulative effects of multisystem damage on mortality risk and provides a new theoretical basis for integrated prevention and precision management of chronic disease [8].

Although several prospective cohort studies have reported significant associations between CKM staging and both all-cause and CVD mortality [[9], [10], [11]], no comprehensive systematic review has yet quantitatively synthesized these relationships across different mortality outcomes. With the global burden of CKM-related conditions continuing to rise [3], consolidating evidence on the link between CKM stages and mortality risk is crucial for guiding early intervention in high-risk populations and optimizing public health decision-making [12].

Accordingly, the present study conducted a systematic review and meta-analysis to quantitatively evaluate the associations between CKM stages and the risks of all-cause, CVD, coronary heart disease (CHD), and stroke mortality. By elucidating the cumulative impact of multisystem comorbidity across CKM stages, this study fills a critical evidence gap and provides an empirical foundation for improving clinical risk stratification and guiding precision prevention strategies in populations at risk.

## 2 Methods

### 2.1 Registration and protocol

This systematic review was prospectively registered in PROSPERO (CRD420251161180) and conducted in accordance with the Meta-analysis of Observational Studies in Epidemiology (MOOSE) guidelines [13]. The manuscript was prepared and reported following the Preferred Reporting Items for Systematic Reviews and Meta-Analyses (PRISMA) statement [14].

### 2.2 Literature search strategy

A comprehensive literature search was conducted on August 8, 2025, using PubMed, Embase, Web of Science, and the Cochrane Library. The search strategy combined Medical Subject Headings (MeSH) and free-text terms related to “cardiovascular–kidney–metabolic syndrome” and mortality outcomes (including all-cause mortality, cardiovascular mortality, coronary heart disease mortality, and stroke mortality).

Because the CKM staging framework was first proposed by the American Heart Association (AHA) in 2023 [6], the search was restricted to studies published from January 1, 2023, to August 8, 2025, to ensure conceptual consistency and relevance. The complete database search strategies are provided in Table S1.

### 2.3 Inclusion and exclusion criteria

#### Inclusion criteria

Population: Adults aged ≥18 years from the general population or community/longitudinal cohorts.

Exposure: Studies explicitly defined cardiovascular-kidney-metabolic (CKM) stages (Stage 0–4) as the primary exposure, adopting the CKM staging framework proposed by the American Heart Association in 2023. Although all studies followed this framework, cohort-specific operational differences existed, mainly in Stage 3 definitions: some studies used direct subclinical cardiovascular biomarkers or imaging, whereas others relied on validated risk-equivalent approaches (e.g., PREVENT or China-PAR risk scores, or very-high-risk chronic kidney disease). Detailed cohort-specific CKM stage definitions are provided in Table S2.

Comparators: Non-CKM participants (Stage 0) as the reference group, or comparisons between different CKM stages.

Outcomes: Studies reporting at least one mortality outcome.

Primary outcomes: All-cause mortality and CVD mortality. Secondary outcomes: CHD mortality and stroke mortality.

Study design: Prospective or retrospective cohort studies.

Data source management: When multiple studies were derived from the same database (e.g., National Health and Nutrition Examination Survey [NHANES]) and assessed identical outcomes, the study with the longest follow-up, largest sample size, and most comprehensive covariate adjustment was retained to avoid double-counting of overlapping populations.

Other criteria: English-language publications released after 2023.

#### Exclusion criteria

Studies were excluded if they: (1) involved participants <18 years or with specific diseases, or examined only a single risk factor without applying the CKM framework; (2) lacked CKM staging, effect estimates, or a defined comparator; (3) reported only incidence data; or (4) were cross-sectional, case series, case reports, RCTs, reviews, commentaries, abstracts, non-English, or unavailable in full text.

### 2.4 Study selection

All retrieved records were imported into EndNote X9, and duplicate records were removed. Two reviewers independently screened titles and abstracts for eligibility, followed by full-text assessment of potentially relevant articles. Discrepancies were resolved through discussion, and if consensus could not be reached, a third reviewer was consulted. The study selection process adhered to PRISMA guidelines, and the detailed screening flow is presented in the PRISMA flow diagram [14,15].

### 2.5 Data extraction

Two reviewers independently extracted data using a standardized predesigned form. Extracted information included study and population characteristics, exposure definitions, covariate adjustments, outcomes, and statistical estimates. Discrepancies or missing information were resolved through discussion and, when necessary, adjudicated by a third reviewer [14,15].

### 2.6 Quality assessment

The methodological quality of the included studies was appraised using the Joanna Briggs Institute (JBI) Critical Appraisal Checklist for Cohort Studies, comprising 11 domains evaluating participant selection, exposure and outcome ascertainment, adequacy of follow-up, and management of confounding. Each item was rated as Yes, No, Unclear, or Not applicable [16]. Two reviewers independently performed the appraisal, and discrepancies were resolved through discussion or consultation with a third reviewer.

### 2.7 Statistical analysis

All analyses were performed using R software (version 4.3.2) with the meta and metafor packages [17]. Pooled hazard ratios (HRs) and 95 % confidence intervals (CIs) were calculated to estimate the associations between CKM stages and the risks of all-cause, CVD, CHD, and stroke mortality. Results were visualized using forest plots. Predictive analyses were performed using the dmetar package to examine risk trends across CKM stages.

Subgroup analyses by sex were conducted for all-cause and CVD mortality to explore potential sex-related differences. Given the potential variation in true effect sizes across studies, random-effects models were employed as the primary analytical approach, in accordance with the recommendations of Borenstein et al. [18].

To explore potential sources of heterogeneity, meta-regression analyses were performed using the following study-level covariates: mean age, proportion of females and males, sample size, mean body mass index (BMI), proportion of physically inactive participants, smokers, drinkers, individuals with education below high school, and calendar period [19]. Covariates showing significant associations (mean age and education level) were further examined through stratified analyses and leave-one-out (LOO) sensitivity analyses to test the robustness of findings [20].

Educational level stratification was based on the median proportion of participants with education below high school (∼50 %), classifying studies into high- and low-education groups. Age stratification was based on the median of mean ages (∼62.5 years), categorizing studies into younger (<62.5 years) and older (≥62.5 years) groups to evaluate potential age-related differences in effect estimates.

LOO sensitivity analyses were conducted to assess the influence of each individual study on the pooled estimates [20]. Baujat plots were generated to identify studies contributing most to heterogeneity, while funnel plots and Egger’s tests were used to assess publication bias [[21], [22], [23]]. Study quality results were visualized using heatmaps based on the JBI cohort checklist to provide an intuitive overview of methodological quality distribution across studies.

## 3 Results

### 3.1 Study selection and characteristics

**Fig. 1:**
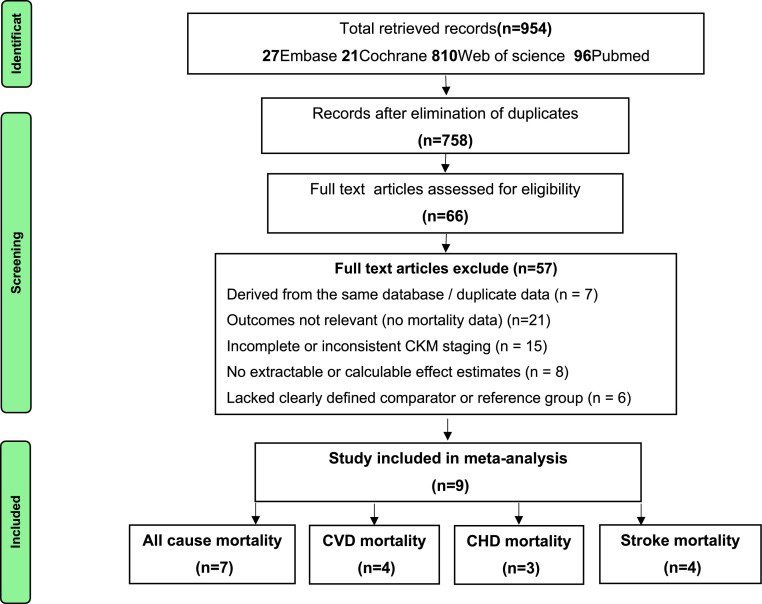
PRISMA Flow Diagram of Study Selection.

According to the predefined search strategy, 954 records were identified. After removing 196 duplicates, 758 unique records remained for title and abstract screening, and 66 articles were retained for full-text assessment. During screening, 7 additional reports derived from overlapping NHANES databases were identified and excluded to prevent duplication of study populations [9,[24], [25], [26], [27], [28], [29]]. Based on the predefined inclusion and exclusion criteria, 9 unique cohort studies were finally included in the quantitative synthesis (Fig. 1) [11,[30], [31], [32], [33], [34], [35], [36], [37]].

All included studies were published within the past three years and predominantly adopted large-scale prospective or retrospective cohort designs, encompassing a combined total of 10,330,498 participants.

In detail, the Chinese cohorts were derived from nationwide health surveys or large community-based databases, including the China Hypertension Survey [30], Cardiometabolic Disease and Cancer Cohort (4C) Study [34], Kailuan Study [11], China Multi-provincial Cohort Study(CMCS) [35] and Northwest China Real-World and Population-based Tianshan cohort (NCRP) [36]. The Taiwan study utilized data from the MJ Health Checkup Database [31], the U.S. studies were based on NHANES continuous cycles [32,33], and the U.K. study drew on data from the UK Biobank [37]. Collectively, these studies provide representative and cross-system evidence of CKM syndrome across diverse populations and healthcare settings.

In all included studies, CKM stages (Stage 0–4) served as the primary exposure variable. The mean age of participants ranged from 40 to 60 years, and the sex distribution was generally balanced. The primary outcomes were all-cause mortality and CVD mortality. The detailed characteristics of the included studies are presented in Table S3.

### 3.2 Association between CKM stages and all-cause mortality

**Fig. 2:**
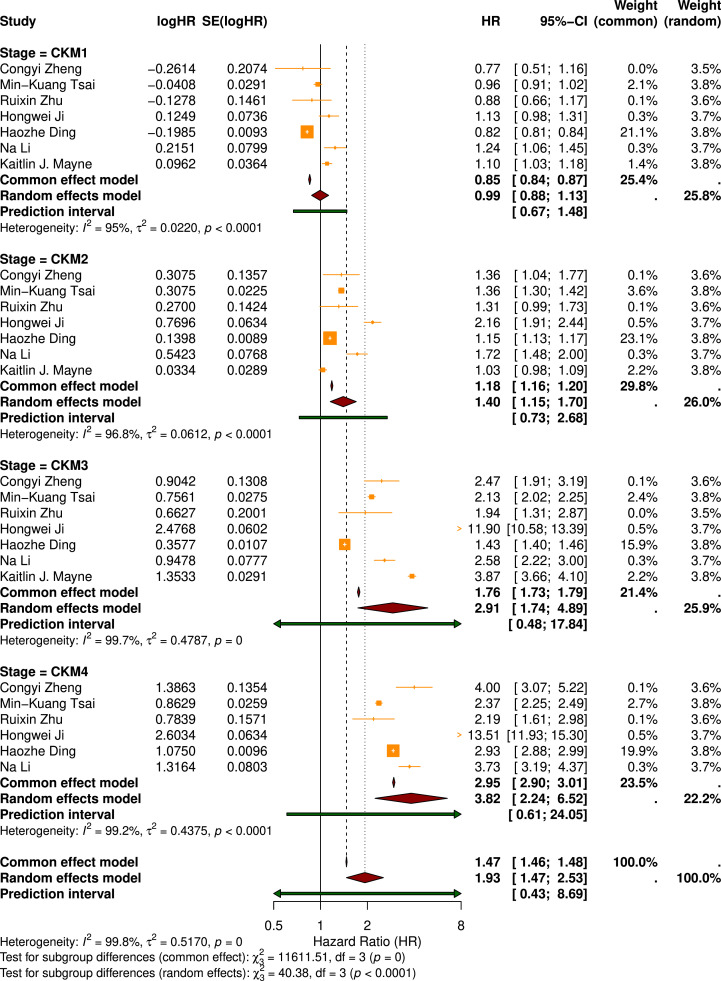
Forest Plot of Pooled Hazard Ratios for All-Cause Mortality Across CKM Stages.

A total of 7 studies, comprising 2424,390 participants, investigated the association between CKM stages and the risk of all-cause mortality. Overall, the random-effects meta-analysis demonstrated a progressive increase in all-cause mortality risk with advancing CKM stages. Compared with Stage 0, significantly higher risks of all-cause mortality were observed in Stage 2 (HR = 1.40, 95 % CI: 1.15–1.70), Stage 3 (HR = 2.91, 95 % CI: 1.74–4.89), and Stage 4 (HR = 3.82, 95 % CI: 2.24–6.52) (Fig. 2). Overall heterogeneity was substantial (I² > 90 %). Prediction intervals displayed in Fig. 2 illustrate the expected range of effects across future populations. Subgroup analyses by sex showed that females consistently exhibited slightly higher risks of all-cause mortality across CKM stages than males (Figure S1).

**Fig. 3:**
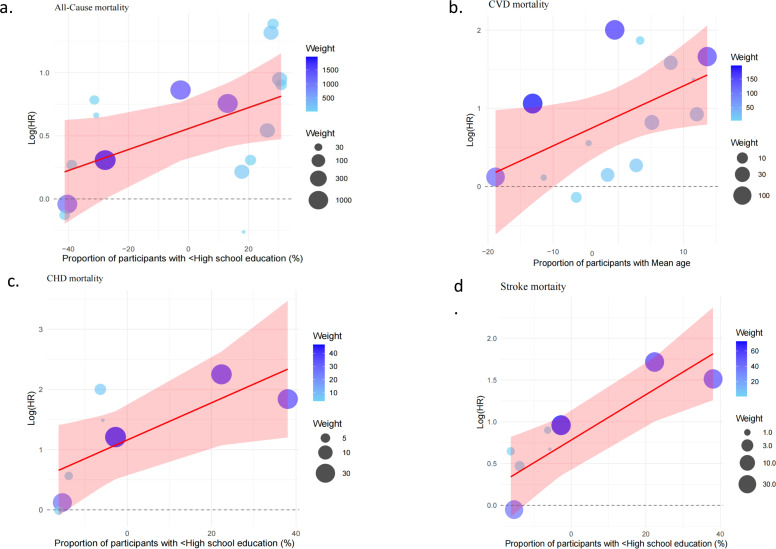
Bubble Plots of Significant Study-Level Moderators Identified by Meta-Regression Analyses.

To explore potential sources of heterogeneity, meta-regression analyses were conducted. None of the examined study-level covariates-including mean age, sex distribution (proportion of females and males), sample size, mean BMI, prevalence of physical inactivity, smoking, drinking, or calendar period—significantly explained the between-study variability (*P* > 0.05; Table S3). In contrast, the proportion of participants with education below high school emerged as a significant moderator (β = 0.0083 per 10 % increase, *P* = 0.047; Table S4, Fig. 3). Inclusion of this covariate reduced between-study variance (τ²) by approximately 24.5 %, indicating that educational attainment partially accounted for the observed heterogeneity.

Given the significant effect of education level in the meta-regression, further subgroup analyses and LOO sensitivity analyses were performed to verify the robustness of this moderating effect. Subgroup analyses revealed that the pooled risk of all-cause mortality was higher in the low-education group (HR=1.98, 95 % CI: 1.4–2.78) than in the high-education group (HR=1.54, 95 % CI: 1.16–2.05; Table S5). The LOO sensitivity analyses further confirmed the robustness of the findings; sequentially removing any single study did not result in significant changes in the pooled estimates (Table S6).

### 3.3 Association between CKM stages and CVD mortality

**Fig. 4:**
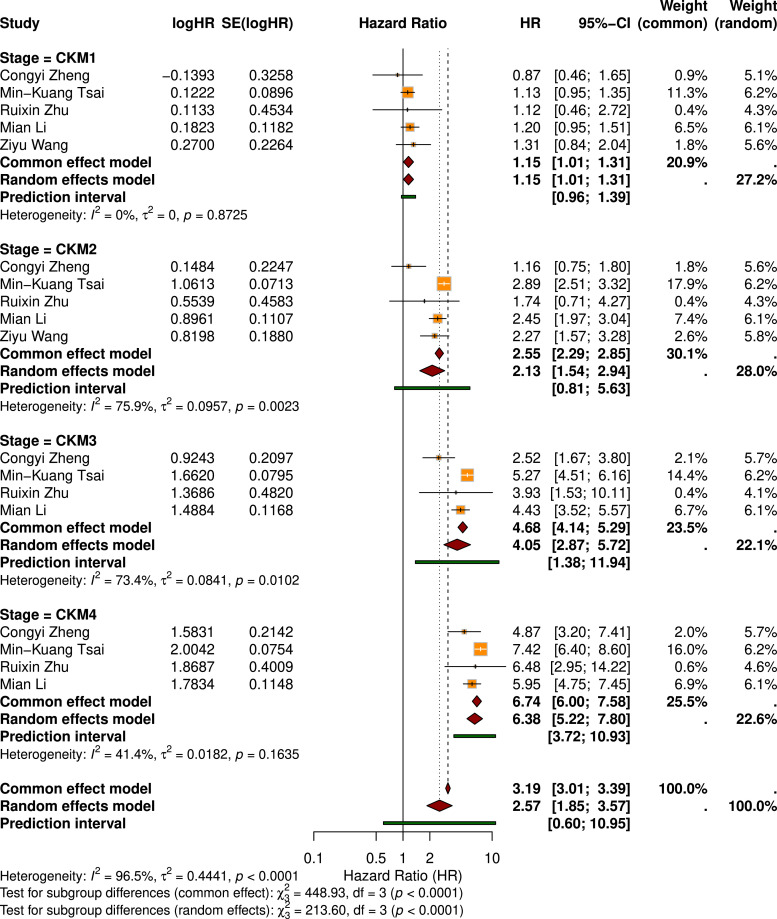
Forest Plot of Pooled Hazard Ratios for Cardiovascular Mortality Across CKM Stages.

Four studies involving 673,214 participants examined the association between CKM stages and CVD mortality. Using random-effects meta-analysis, significantly higher risks were observed compared with Stage 0 for Stage 1 (HR = 1.15, 95 % CI: 1.01–1.31), Stage 2 (HR = 2.13, 95 % CI: 1.54–2.94), Stage 3 (HR = 4.05, 95 % CI: 2.87–5.72), and Stage 4 (HR = 6.38, 95 % CI: 5.22–7.80), demonstrating a clear dose–response pattern (Fig. 4). Overall heterogeneity was moderate (I² < 75 %). Prediction intervals displayed in Fig. 4 showed consistent directional trends across stages, supporting the robustness of the pooled estimates. Sex-stratified subgroup analyses revealed stage-specific differences (Figure S2), with females exhibiting higher risk at Stage 2, males at Stage 3, and both sexes reaching the highest risk at Stage 4.

Meta-regression identified mean age as a significant moderator of CVD mortality (β = 0.0383 per 1-year increase, *P* = 0.046; Table S4, Fig. 3). Inclusion of mean age as a covariate reduced between-study variance (τ²) by approximately 22.47 %. Age subgroup analyses showed a higher pooled risk in the high-age group (HR=3.29, 95 % CI: 2.10–5.14) than in the low-age group (HR=1.79, 95 % CI: 0.90–3.56; Table S5). LOO sensitivity analyses confirmed the robustness of these results (Table S6).

### 3.4 Secondary cardiovascular outcomes (CHD mortality and stroke mortality)

The random-effects meta-analysis demonstrated a clear progressive increase in the risks of CHD and stroke mortality across CKM stages (Figures S3-S4). Compared with Stage 0, no significant elevation was observed in Stage 1 (CHD: HR = 1.11, 95 % CI: 0.83–1.50; stroke: HR = 1.16, 95 % CI: 0.91–1.47). However, risks increased markedly from Stage 2 (CHD: HR = 2.55, 95 % CI: 1.67–3.91; stroke: HR = 2.11, 95 % CI: 1.53–2.91) and further escalated in Stage 3 (CHD: HR = 6.10, 95 % CI: 4.51–8.26; stroke: HR = 6.29, 95 % CI: 2.54–15.56), reaching the highest levels at Stage 4 (CHD: HR = 9.19, 95 % CI: 6.93–12.20; stroke: HR = 5.48, 95 % CI: 4.28–7.02).

Prediction intervals shown in Figures S3-S4 indicate that the direction and magnitude of associations were generally consistent across potential future populations.

Meta-regression analysis further revealed that a higher proportion of participants with low educational attainment was significantly associated with increased CHD mortality (β=0.031 per 10 % increase, *P* = 0.035) and stroke mortality (β=0.027 per 10 % increase, *P* = 0.0046) (Table S4, Fig. 3). Inclusion of this covariate reduced between-study variance (τ²) by 54.8 % for CHD and 67.6 % for stroke mortality, indicating that educational attainment substantially accounted for the observed heterogeneity. Stratified analyses confirmed this finding: pooled risk estimates were higher in the low-education subgroup (CHD: HR=1.45, 95 % CI: 1.20–1.70; Stroke: HR=1.38, 95 % CI: 1.16–1.62) than in the high-education subgroup (CHD: HR=1.12, 95 % CI: 0.94–1.33; Stroke: HR=1.10, 95 % CI: 0.91–1.32) (Table S5). LOO sensitivity analyses indicated that the overall results were robust (Table S6).

### 3.5 Sensitivity analyses and publication bias

**Fig. 5:**
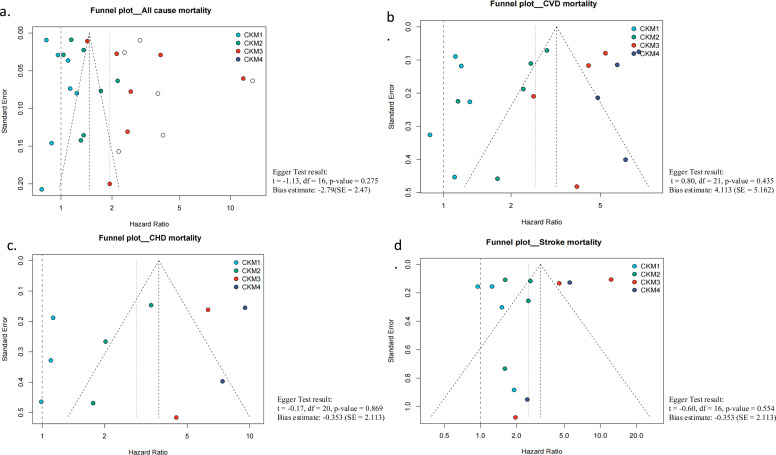
Funnel Plots for Assessing Publication Bias Across Mortality Outcomes.

LOO sensitivity analyses (Table S7) and Baujat plots (Table S8, Figure S5) consistently confirmed the stability of the pooled estimates for all-cause, CVD, CHD, and stroke mortality outcomes. Exclusion of any single study did not materially alter the direction or significance of the summary HRs, indicating that no individual study exerted undue influence on the overall effect estimates. Egger’s regression tests detected no evidence of small-study effects or publication bias (*P* > 0.05), and funnel plots appeared symmetrical, supporting the reliability and robustness of the pooled findings (Fig. 5).

### 3.6 Methodological quality of included studies

The methodological quality of the included studies, assessed using the JBI Critical Appraisal Checklist for Cohort Studies, was generally high. All studies used consistent CKM staging criteria, valid exposure and outcome assessments, and multivariable models to control confounding. A few studies lacked detailed reporting on follow-up completeness and missing data handling, leading to isolated Unclear ratings. Overall, the risk of bias was considered low (Table S9, FigureS6).

## 4 Discussion

This study, based on large-scale prospective and retrospective cohorts from multiple countries, is the first systematic review and meta-analysis to comprehensively quantify the associations between the stages of CKM syndrome and multidimensional mortality risks. The findings demonstrated a consistent, progressive increase in all-cause, CVD, CHD, and stroke mortality with advancing CKM stages. Compared with Stage 0, individuals at Stages 2, 3, and 4 exhibited 1.40, 2.91, and 3.82 fold higher risks of all-cause mortality, respectively; the risks of cardiovascular mortality increased to 2.13, 4.05, and 6.38 fold; and the risks of CHD and stroke mortality showed the steepest rise at the advanced stage (Stage 4: HR = 9.19 and 5.48, respectively). Consistent with several large-scale cohort studies, our findings further confirm a stepwise gradient in mortality risk across CKM stages and highlight the potential of CKM staging as a robust predictor of long-term mortality [6,11,35].

This study also revealed significant sex-specific differences in the association between CKM stages and mortality risk, a pattern highly consistent with findings from several large cohort studies [32,33,37]. Women demonstrated generally higher all-cause mortality across CKM stages, suggesting that systemic risk elevation may occur earlier during the onset of metabolic and renal dysfunction. This disparity likely reflects estrogen decline after menopause, which accelerates metabolic, renal, and vascular dysfunction [38 39]. In contrast, men exhibited a steeper rise in cardiovascular mortality during the mid-to-late stages of CKM progression, potentially due to higher prevalence of hypertension, smoking, alcohol consumption, and visceral adiposity, which contribute to a clustering of cardiovascular events in the later disease stages [40,41]. Highlight the importance of incorporating sex-specific characteristics into risk evaluation and disease surveillance to improve the precision of CKM-related risk stratification.

Beyond sex differences, this study also identified age as a significant moderator of the association between CKM stages and CVD mortality. Meta-regression analyses indicated that for each 1-year increase in the mean age of participants, the logarithm of the hazard ratio (log HR) for CVD mortality increased by approximately 0.038. Mean age explained approximately 22 % of the between-study heterogeneity, suggesting that the impact of CKM staging on cardiovascular mortality becomes stronger with advancing age. This pattern may reflect the cumulative impact of age-related metabolic dysregulation, renal decline, and vascular remodeling, which collectively amplify the interconnected pathophysiology of CKM and underscore the importance of integrating aging mechanisms into future risk assessment frameworks.

This study also revealed a significant moderating effect of educational attainment on the association between CKM stages and mortality outcomes. Meta-regression analyses showed that for every 10 % increase in the proportion of individuals with low educational attainment, the log HR increased by 0.008 for all-cause mortality, 0.031 for CHD mortality, and 0.027 for stroke mortality. Educational attainment accounted for approximately 25 %, 55 %, and 68 % of the between-study heterogeneity for these outcomes, respectively, indicating that socioeconomic composition substantially contributes to variability in mortality estimates. The observed gradient may partly reflect cumulative socioeconomic disadvantage [42,43]—manifested through differences in health literacy, access to care, and chronic exposure to metabolic and behavioral stressors [[44], [45], [46]]—that accelerates CKM progression and amplifies mortality risk.

Notably, heterogeneity for all-cause mortality was substantial (I² > 90 %), which is expected when synthesizing very large population-based cohorts from different regions and healthcare systems that operationalized CKM staging with partially different inputs. Several factors likely contributed to this variability. First, although all studies adopted the AHA 2023 CKM framework, the definition of CKM Stage 3 varied most across cohorts—some used direct subclinical cardiovascular markers or imaging, whereas others relied on validated risk-equivalent approaches (e.g., PREVENT/China-PAR scores or very-high-risk CKD). Such differences can shift participants across adjacent stages and alter the apparent stage–mortality gradient. Second, baseline risk profiles differed markedly across cohorts (age structure, cardiometabolic risk burden, CKD ascertainment, and event rates), which can lead to genuine between-study differences in effect sizes even under a shared conceptual framework. Third, the extent of confounding control and outcome ascertainment (e.g., registry linkage vs self-report, and variability in follow-up duration) may have further amplified dispersion in estimates. Importantly, multiple analyses supported the robustness of the overall direction of association despite heterogeneity. Prediction intervals consistently indicated a positive association across potential future populations, and leave-one-out and Baujat diagnostics suggested that no single study disproportionately drove the pooled effects. Moreover, meta-regression identified educational attainment as a significant contributor to between-study variability for all-cause mortality, indicating that heterogeneity in sociodemographic composition partially underlies the observed inconsistency. Taken together, the substantial I² is more likely to reflect real-world diversity in CKM operationalization and population risk contexts, rather than instability of the underlying association.

Overall, this study highlights the biological and sociodemographic heterogeneity underlying the relationship between CKM stages and mortality. Sex, age, and education independently and interactively shape risk accumulation across CKM stages, underscoring that CKM represents a multidimensional network driven by biological, behavioral, and social determinants rather than a single disease continuum.

## Strengths and limitations

This study has several notable strengths. First, it represents the first comprehensive meta-analysis to systematically integrate evidence on the association between the stages of the CKM syndrome and multidimensional mortality risks. By pooling large-scale population-based data from multiple countries and databases, the present study substantially enhances the representativeness and external validity of its findings. Second, the study provides methodological validation of the CKM framework, offering the first quantitative evidence supporting its prognostic value for risk stratification, thereby laying a foundation for the development of CKM-based predictive tools. Third, it introduces analytical innovation and multidimensionality: by simultaneously quantifying all-cause, cardiovascular, coronary, and stroke mortality and exploring the moderating roles of key variables such as sex, age, and education level, the study elucidates both the biological and sociodemographic heterogeneity inherent in CKM progression. Finally, the research employed rigorous quality control and robustness verification procedures—including quality assessment using the JBI tool, random-effects pooling, meta-regression, subgroup and sensitivity analyses—to ensure the reliability and interpretability of the results.

However, several limitations should be acknowledged. First, because the CKM concept was only recently proposed by the American Heart Association in 2023, the number of eligible studies remains limited. Most available studies are still in an early exploratory phase, and partial overlap in database sources may have introduced uncertainty into the pooled estimates. Second, despite all included studies being aligned with the AHA 2023 CKM framework, operational definitions of specific stages—particularly CKM Stage 3—varied across cohorts. Differences in the use of subclinical cardiovascular markers, imaging-based assessments, or risk-equivalent algorithms may have led to stage misclassification and contributed to between-study heterogeneity. Third, variability in covariate adjustment across studies, particularly incomplete control for behavioral, pharmacological, or inflammatory confounders, may have led to residual confounding. Fourth, given the observational nature of the included data, causal inference remains limited, and the directionality of risk should be interpreted with caution. Lastly, unmeasured socioeconomic and regional disparities may have contributed to variation in overall risk estimates, underscoring the need for further validation across diverse populations and healthcare settings.

To enhance the clinical utility of the CKM staging framework, future studies should move beyond traditional risk factor analysis by objectively integrating detailed metrics of physical activity and key lifestyle behaviors. Clarifying the extent to which lifestyle factors can mitigate the cumulative mortality risk across different CKM stages will inform subsequent research. This integrated approach can guide the development of precise, evidence-based exercise and public health interventions.

## Conclusion

This systematic review and meta-analysis of large population-based cohorts across multiple countries confirmed a significant, stage-dependent gradient between CKM stages and the risks of all-cause, CVD, CHD, and stroke mortality. Moreover, modifiers such as sex, age, and educational attainment substantially influence these associations, underscoring the need to incorporate both biological and social determinants into risk assessment and prevention strategies for more precise control of CKM-related diseases.

## Article information

### Support

None.

### Financial disclosure

The authors declare that they have no relevant financial interests.

### Data sharing

All extracted and calculated data are available from the corresponding author upon reasonable request.

### Ethics approval

Ethical approval was not required for this study because it was based on published data and did not involve individual participant information.

## Abbreviations and acronyms

CKM: Cardiovascular–Kidney–Metabolic, CKM stages: Stages 0–4 of the Cardiovascular–Kidney–Metabolic framework, AHA: American Heart Association, BMI: Body Mass Index, CHD: Coronary Heart Disease, CI: Confidence Interval, CKD: Chronic Kidney Disease, CVD: Cardiovascular Disease, HR: Hazard Ratio, JBI: Joanna Briggs Institute, LOO: Leave-One-Out, MetS: Metabolic Syndrome, MOOSE: Meta-analysis of Observational Studies in Epidemiology, NHANES: National Health and Nutrition Examination Survey, PRISMA: Preferred Reporting Items for Systematic Reviews and Meta-Analyses, PROSPERO: International Prospective Register of Systematic Reviews.

**Figure:**
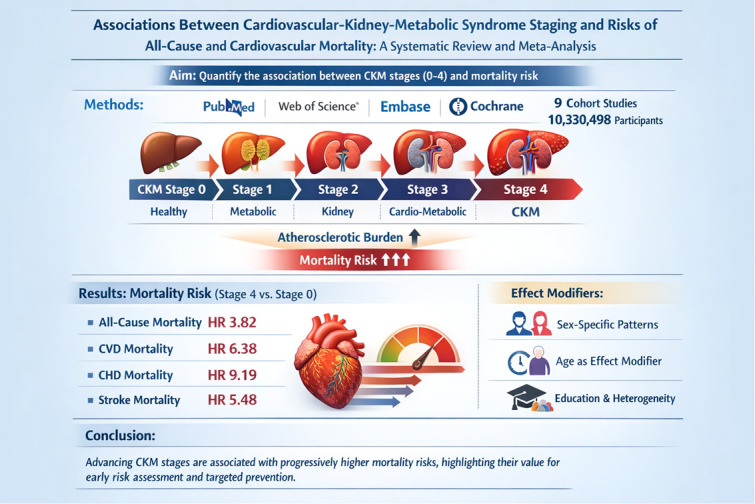
Central illustration.

## Author declaration template

We wish to draw the attention of the Editor to the following facts which may be considered as potential conflicts of interest and to significant financial contributions to this work. [OR] We wish to confirm that there are no known conflicts of interest associated with this publication and there has been no significant financial support for this work that could have influenced its outcome.

We confirm that the manuscript has been read and approved by all named authors and that there are no other persons who satisfied the criteria for authorship but are not listed. We further confirm that the order of authors listed in the manuscript has been approved by all of us.

We confirm that we have given due consideration to the protection of intellectual property associated with this work and that there are no impediments to publication, including the timing of publication, with respect to intellectual property. In so doing we confirm that we have followed the regulations of our institutions concerning intellectual property.

We further confirm that any aspect of the work covered in this manuscript that has involved either experimental animals or human patients has been conducted with the ethical approval of all relevant bodies and that such approvals are acknowledged within the manuscript. [CAN BE DELETED IF NOT RELEVANT]

We understand that the Corresponding Author is the sole contact for the Editorial process (including Editorial Manager and direct communications with the office). He/she is responsible for communicating with the other authors about progress, submissions of revisions and final approval of proofs. We confirm that we have provided a current, correct email address which is accessible by the Corresponding Author.

Signed by all authors as follows:

[LIST AUTHORS AND DATED SIGNATURES ALONGSIDE]

## CRediT authorship contribution statement

**Huimin Ding:** Writing – original draft, Visualization, Methodology, Investigation, Formal analysis, Data curation, Conceptualization. **Liqun Jiang:** Writing – review & editing, Investigation, Formal analysis, Data curation. **Yiqiu Zhao:** Writing – review & editing, Validation, Methodology. **Dongjun Lee:** Writing – review & editing, Validation, Methodology. **Buongo Chun:** Writing – review & editing, Supervision, Project administration, Conceptualization.

## Declaration of competing interest

The authors declare that they have no known competing financial interests or personal relationships that could have appeared to influence the work reported in this paper.

## Appendix Supplementary materials

Image, application 1
