# Supplementary material for: Associations between cardiovascular-kidney-metabolic syndrome staging and risks of all-cause and cardiovascular mortality: a systematic review and meta-analysis
Source: Am J Prev Cardiol. 2026 Jan 2;25:101407. doi: 10.1016/j.ajpc.2026.101407 (PMC12811431; doi:10.1016/j.ajpc.2026.101407)

**Supplementary Appendix**

**[Appendix 1:Methods 1](#_Toc2107659540)**

[Table S1. Search Strategies Per Database 1](#_Toc691131515)

[Table S2.Operational definitions of cardiovascular–kidney–metabolic (CKM) stages across included cohorts. 5](#_Toc108325982)

**[Appendix 2:Results 7](#_Toc1716130465)**

[Table S3. Characteristics of included studies. 7](#_Toc151862398)

[Figure S1. Forest plot of all-cause mortality by CKM stage and sex 9](#_Toc1140750550)

[Figure S2. Forest plot of CVD mortality by CKM stage and sex 10](#_Toc2007977081)

[Figure S3. Forest plot of CHD mortality by CKM stage 11](#_Toc365287762)

[Figure S4. Forest plot of stroke mortality by CKM stage 12](#_Toc1883152808)

[Table S4. Meta-regression analyses of study-level moderators for all-cause, CVD, CHD, and stroke mortality 13](#_Toc535254570)

[Table S5. Subgroup meta-analysis for covariates showing significant differences in meta-regression (education level and mean age) 16](#_Toc214560707)

[Table S6. Leave-One-Out sensitivity analyses for covariates showing significant differences in meta-regression (education level and mean age) 17](#_Toc496759236)

[Table S7. All Outcomes Leave-One-Out Sensitivity Analysis 19](#_Toc1763543563)

[Figure S5. Baujat plot 23](#_Toc307367447)

[Table S8. Baujat Sensitivity Analysis Across All Outcomes 25](#_Toc1226510694)

[Table S9. Methodological quality of included cohort studies based on the JBI Critical Appraisal Checklist 27](#_Toc269706505)

[Figure S6. JBI Quality Appraisal Heatmap 32](#_Toc1766734365)

# Appendix 1:Methods

## **Table S1. Search Strategies Per Database**

| **Database: PubMed <8 August 2025>** | |
| --- | --- |
| **#1** | "Cardiovascular-Kidney-Metabolic Syndrome"[Title/Abstract] OR "CKM syndrome"[Title/Abstract] OR "cardiometabolic syndrome with kidney disease"[Title/Abstract] OR "cardiorenal metabolic syndrome"[Title/Abstract]  **Results: 410** |
| **#2** | ("Mortality"[MeSH Terms] OR "all-cause mortality"[Title/Abstract] OR "total mortality"[Title/Abstract] OR "overall mortality"[Title/Abstract] OR "cardiovascular mortality"[Title/Abstract] OR "CVD mortality"[Title/Abstract] OR "heart disease mortality"[Title/Abstract] OR "cardiac mortality"[Title/Abstract] OR "coronary heart disease mortality"[Title/Abstract] OR "CHD mortality"[Title/Abstract] OR "ischemic heart disease mortality"[Title/Abstract] OR "stroke mortality"[Title/Abstract] OR "cerebrovascular mortality"[Title/Abstract] OR "fatality"[Title/Abstract] OR "death"[Title/Abstract])  **Results: 1,439, 770** |
| **#3** | ("Cardiovascular-Kidney-Metabolic Syndrome"[Title/Abstract] OR "CKM syndrome"[Title/Abstract] OR "cardiometabolic syndrome with kidney disease"[Title/Abstract] OR "cardiorenal metabolic syndrome"[Title/Abstract]) AND (("Mortality"[MeSH Terms] OR "all-cause mortality"[Title/Abstract] OR "total mortality"[Title/Abstract] OR "overall mortality"[Title/Abstract] OR "cardiovascular mortality"[Title/Abstract] OR "CVD mortality"[Title/Abstract] OR "heart disease mortality"[Title/Abstract] OR "cardiac mortality"[Title/Abstract] OR "coronary heart disease mortality"[Title/Abstract] OR "CHD mortality"[Title/Abstract] OR "ischemic heart disease mortality"[Title/Abstract] OR "stroke mortality"[Title/Abstract] OR "cerebrovascular mortality"[Title/Abstract] OR "fatality"[Title/Abstract] OR "death"[Title/Abstract]))  **Results: 96** |

| **Database: Web of Science <8 August 2025>** | |
| --- | --- |
| **#1** | TS=("Cardiovascular-Kidney-Metabolic Syndrome" OR "CKM syndrome" OR "cardiometabolic syndrome with kidney disease" OR "cardiorenal metabolic syndrome" OR ("cardiometabolic syndrome" AND "chronic kidney disease") OR ("metabolic syndrome" AND "chronic kidney disease") OR "cardiometabolic multimorbidity") and Preprint Citation Index (Exclude – Database) and Research Commons (Exclude – Database)  **Results: 5, 020** |
| **#2** | TS=("all-cause mortality" OR "total mortality" OR "overall mortality" OR "cardiovascular mortality" OR "CVD mortality" OR "heart disease mortality" OR "cardiac mortality" OR "coronary heart disease mortality" OR "CHD mortality" OR "ischemic heart disease mortality" OR "ischaemic heart disease mortality" OR "stroke mortality" OR "cerebrovascular mortality" OR "fatality" OR "death") and Preprint Citation Index (Exclude – Database) and Research Commons (Exclude – Database)  **Results: 2, 202, 898** |
| **#3** | #1 AND #2 and Preprint Citation Index (Exclude – Database) and Research Commons (Exclude – Database)  **Results: 810** |

| **Database: The [Cochrane Library](https://www.sciencedirect.com/topics/biochemistry-genetics-and-molecular-biology/cochrane-library) < 8 August 2025>** | |
| --- | --- |
| **#1** | "Cardiovascular-Kidney-Metabolic Syndrome":ti,ab,kw OR "CKM syndrome":ti,ab,kw OR "cardiometabolic syndrome with kidney disease":ti,ab,kw  OR "cardiorenal metabolic syndrome":ti,ab,kw OR ("cardiometabolic syndrome":ti,ab,kw AND "chronic kidney disease":ti,ab,kw) OR ("metabolic syndrome":ti,ab,kw AND "chronic kidney disease":ti,ab,kw) OR "cardiometabolic multimorbidity":ti,ab,kw  **Results: 106** |
| **#2** | "all-cause mortality":ti,ab,kw OR "total mortality":ti,ab,kw OR "overall mortality":ti,ab,kw OR "cardiovascular mortality":ti,ab,kw OR "CVD mortality":ti,ab,kw OR "heart disease mortality":ti,ab,kw OR "cardiac mortality":ti,ab,kw OR "coronary heart disease mortality":ti,ab,kw OR "CHD mortality":ti,ab,kw OR "ischemic heart disease mortality":ti,ab,kw OR "stroke mortality":ti,ab,kw OR "cerebrovascular mortality":ti,ab,kw OR "fatality":ti,ab,kw OR "death":ti,ab,kw  **Results: 101901** |
| **#3** | #1 and #2  **Results: 21** |

| **Database: Embase< 8 August 2025>** | |
| --- | --- |
| **#1** | 'cardiovascular kidney metabolic syndrome'/exp  **Results: 78** |
| **#2** | 'cardiovascular kidney metabolic syndrome'/exp OR 'cardiometabolic syndrome'/exp) AND 'chronic kidney disease'/exp OR 'cardiorenal syndrome'/exp) AND 'metabolic syndrome'/exp OR 'cardiovascular-kidney-metabolic syndrome':ti,ab,kw OR 'ckm syndrome':ti,ab,kw OR 'cardiometabolic syndrome with kidney disease':ti,ab,kw OR 'cardiorenal metabolic syndrome':ti,ab,kw OR 'cardiometabolic multimorbidity':ti,ab,kw  **Results: 888** |
| **#3** | 'mortality'/exp  **Results: 1,632,127** |
| **#4** | 'mortality'/exp OR 'cardiovascular mortality'/exp OR 'heart disease mortality' OR 'coronary heart disease mortality' OR 'stroke mortality' OR 'all-cause mortality':ti,ab,kw OR 'total mortality':ti,ab,kw OR 'overall mortality':ti,ab,kw OR 'cardiovascular mortality':ti,ab,kw OR 'cvd mortality':ti,ab,kw OR 'chd mortality':ti,ab,kw OR 'ischemic heart disease mortality':ti,ab,kw OR 'ischaemic heart disease mortality':ti,ab,kw OR 'cerebrovascular mortality':ti,ab,kw OR fatality:ti,ab,kw OR death:ti,ab,kw  **Results:2,810,087** |
| **#5** | #1 AND #2  **Results: 76** |
| **#6** | #3 AND #4  **Results: 1,632,127** |
| **#7** | #5 AND #6  **Results: 27** |

## Table S2.Operational definitions of cardiovascular–kidney–metabolic (CKM) stages across included cohorts.

**Purpose: This table summarizes cohort-specific operational definitions of CKM stages to allow transparent assessment of staging comparability and pooling validity. Differences primarily reflect data availability rather than conceptual deviations from the 2023 AHA CKM framework.**

| **First Author (Year)** | **CKM Framework Cited** | **Stages 0–2: Metabolic / Renal Criteria** | **Stage 3: Subclinical CVD Definition (Key Source of Heterogeneity)** | **Stage 4: Clinical CVD Definition** | **Key Deviations / Data Availability Limitations** |
| --- | --- | --- | --- | --- | --- |
| Zheng et al., 2025 | AHA 2023 CKM framework | Obesity and metabolic risk factors defined using Asian-specific BMI and waist circumference; CKD defined by eGFR | Subclinical CVD identified using echocardiographic structural or functional abnormalities; Stages 3 and 4 combined as “advanced CKM” | Included within advanced CKM | Stage 3 and 4 combined due to study design; Asian anthropometric cut-offs |
| Tsai et al., 2025 | AHA 2023 CKM framework (Asian cut-offs) | Stage 1–2 based on BMI, fasting glucose, metabolic risk factors, and CKD (eGFR-based) | Risk-equivalent definition: PREVENT 10-year CVD risk >20% or eGFR ≤30 mL/min/1.73 m² | Self-reported medication use or healthcare-seeking for CHD, HF, or stroke | HbA1c unavailable; proteinuria assessed by dipstick rather than ACR; clinical CVD self-reported |
| Zhu et al., 2025 | AHA 2023 CKM framework | Comparable metabolic criteria; CKD categorized according to KDIGO | Risk-equivalent definition: very-high-risk CKD or elevated PREVENT-predicted CVD risk | Self-reported physician-diagnosed CVD | Lack of imaging and cardiac biomarkers may underestimate Stage 3 |
| Ji et al., 2025 | AHA 2023 CKM framework (NHANES-adapted) | Comparable metabolic criteria; CKD categorized according to KDIGO | Risk-equivalent definition: very-high-risk CKD or elevated PREVENT-predicted CVD risk | Self-reported physician-diagnosed CVD | Limited subclinical CVD phenotyping in NHANES |
| Li et al., 2025 | AHA 2023 CKM framework combined with Life’s Essential 8 | Detailed metabolic and renal assessment including HbA1c and ACR | Defined as presence of subclinical CVD or risk equivalents, operationalized primarily using PREVENT risk scores | Adjudicated clinical CVD (MI, stroke, PAD, CVD death) | Overlap between early CKM stages and LE8 cardiovascular health metrics; PREVENT extrapolation in older adults |
| Wang et al., 2025 | AHA 2023 CKM health metrics and stages | Asian-specific BMI thresholds; CKD defined by eGFR without UACR | Subclinical HF identified using NT-proBNP or hs-troponin T, assessed only in a Beijing sub-cohort | Adjudicated CHD and stroke events | Subclinical atherosclerotic CVD not assessed; Stage 3 limited to HF phenotypes |
| Li et al., 2024 | AHA 2023 CKM framework | Metabolic risk factors and moderate-to-high-risk CKD | Risk-equivalent definition: China-PAR–predicted 10-year CVD risk ≥20% or very-high-risk CKD | Clinically diagnosed CVD confirmed via electronic health records | Imaging unavailable; Stage 3 defined via risk equivalence |
| Ding et al., 2025 | AHA 2023 CKM framework | Stage 1–2 defined by metabolic risk factors and CKD | Risk-equivalent definition: China-PAR 10-year CVD risk ≥20% or very-high-risk CKD | Follow-up–ascertained clinical CVD; subdivided into CKD vs non-CKD CVD | Preprint; reliance on risk prediction |
| Mayne et al., 2025 | AHA 2023 CKM framework | Metabolic risk factors and CKD | Risk-equivalent definition: subclinical CVD defined using predicted CVD risk (PREVENT) | Clinically diagnosed CVD | Limited reporting of imaging or biomarker-based subclinical CVD |

Across cohorts, CKM stages 0–2 were defined using largely comparable metabolic and renal criteria. In contrast, substantial heterogeneity was observed in Stage 3 definitions, reflecting differences in the availability of subclinical cardiovascular phenotyping. Some studies identified Stage 3 using direct biomarkers or imaging, whereas others relied on validated risk-equivalent approaches (e.g., PREVENT or China-PAR scores, or very-high-risk CKD). Stage 4 definitions also varied with respect to outcome ascertainment methods. These operational differences were considered in the interpretation of pooled estimates.

# Appendix 2:Results

## Table S3. Characteristics of included studies.

| **First Author (Year)** | **Country** | **Study Design & Data Source** | **Sample Size (n)** | **Mean Age (years) / Sex (%)** | **CKM Definition / Staging Method** | **Main Outcomes** | **Key Findings** |
| --- | --- | --- | --- | --- | --- | --- | --- |
| Zheng et al., 2025 | China | Nationally representative retrospective cohort (China Hypertension Survey, 2012–2015; median follow-up 5 years) | 33,685 | 57.1; 57.8% female | AHA 2023 CKM Stages 0–4; cardiac structural indicators (echocardiography), eGFR, and metabolic parameters | All-cause and CVD mortality | Higher CKM stages associated with progressively increased mortality; HR for Stage 4 ≈ 2.5 vs Stage 0 after full adjustment. |
| Tsai et al., 2025 | Taiwan | Retrospective cohort from MJ Health Screening Program (1996–2017), follow-up through 2020 | 515,602 | 40.3; 49.9% female | AHA 2023 CKM Stages (Asian BMI ≥23 kg/m²; waist ≥80/90 cm) | All-cause, CVD, and renal mortality; end-stage kidney disease (ESKD) | CKM Stage ≥2 contributed to >30% of total mortality; risk increased steadily with stage; consistent across sex and age subgroups. |
| Zhu et al., 2025 | United States | Prospective NHANES 1999–2018, linked to National Death Index, follow-up to 2019 | 27,909 | 49.7; 49% female | AHA 2023 CKM Stages; Stage 3 defined using PREVENT 10-year ASCVD risk equation | Premature all-cause and CVD mortality (<75 y) | Higher CKM stages significantly associated with premature mortality; social determinants of health (SDOHs) modified risk strength. |
| Ji et al., 2025 | United States | Prospective national cohort (NHANES 1988–2018); median follow-up 13.3 years | 33,868 | 48.4; 52% female | AHA 2023 CKM Stages (adapted for NHANES biomarker availability) | All-cause and CVD mortality | CKM Stage ≥2 markedly elevated mortality risk (HR 1.3–3.5); stronger relative risk in females; consistent across race groups. |
| Li et al., 2025 | China | Multicenter prospective cohort (4C-China Cardiometabolic and Cancer Cohort); median follow-up 10.1 years | 100,727 | ≥40 y; balanced sex | CKM Stages 0–4 combined with Life’s Essential 8 (LE8) cardiovascular health score | Composite CVD events (MI, stroke, CVD death) | CKM staging strongly associated with higher CVD event risk; good LE8 scores attenuated this risk by 30–40%. |
| Wang et al., 2025 | China | Community-based prospective cohort (CMCS main + Beijing sub-cohort) | 5,293 + 2,295 | 61.1; 47.8% male | CKM health metrics and stages (0–2 main cohort; 3 in sub-cohort) | 10-year incident CVD risk (CHD, stroke, HF) | CVD incidence rose with CKM stage; model including CKM outperformed traditional metabolic risk factors. |
| Li et al., 2024 | China | Prospective community cohort (Kailuan Study), follow-up through 2021 | 97,777 | 51.2; 77.8% male | AHA CKM Stages 0–4; Stage 4 subdivided into 4a (CVD without CKD) and 4b (CVD with CKD) | All-cause mortality | Stepwise risk gradient observed; Stage 4b exhibited the highest mortality (HR ≈ 5.9). |
| Ding et al., 2025 | China | Northwestern China Health Examination Cohort (2019–2023) using multi-state Markov modeling | 9,116,728 | 49.0; 53% female | AHA CKM Stages 0–4 based on blood pressure, glucose, lipids, eGFR, and CVD history | All-cause mortality; stage-transition probability | CKM Stage ≥ 3 substantially shortened life expectancy; high transition rates observed between adjacent stages. |
| Mayne et al., 2025 | United Kingdom | Prospective UK Biobank cohort (baseline 2007–2010; follow-up to 2025) | 404,202 | 57 ± 8; 54% female | AHA 2023 CKM Stages (Stage 3 defined by KDIGO CKD criteria and PREVENT risk) | All-cause and CVD mortality | Over 13.7 years’ median follow-up, CKM Stage ≥ 2 strongly predicted mortality (HR ≈ 1.3–6.9). Sex-stratified analysis showed higher relative risk in females but greater absolute mortality in males. |

**Abbreviations:**AHA, American Heart Association; ASCVD, Atherosclerotic Cardiovascular Disease; BMI, Body Mass Index; CKD, Chronic Kidney Disease; CKM, Cardiovascular–Kidney–Metabolic Syndrome; CMCS, China Metabolic and Cardiovascular Community Study; CVD, Cardiovascular Disease; eGFR, Estimated Glomerular Filtration Rate; ESKD, End-Stage Kidney Disease; HF, Heart Failure; HR, Hazard Ratio; KDIGO, Kidney Disease: Improving Global Outcomes; LE8, Life’s Essential 8; MI, Myocardial Infarction; NHANES, National Health and Nutrition Examination Survey; PREVENT, Prediction Equations for Cardiovascular Risk; SDOHs, Social Determinants of Health; uACR, Urinary Albumin-to-Creatinine Ratio; UK Biobank, United Kingdom Biobank.

## Figure S1. Forest plot of all-cause mortality by CKM stage and sex


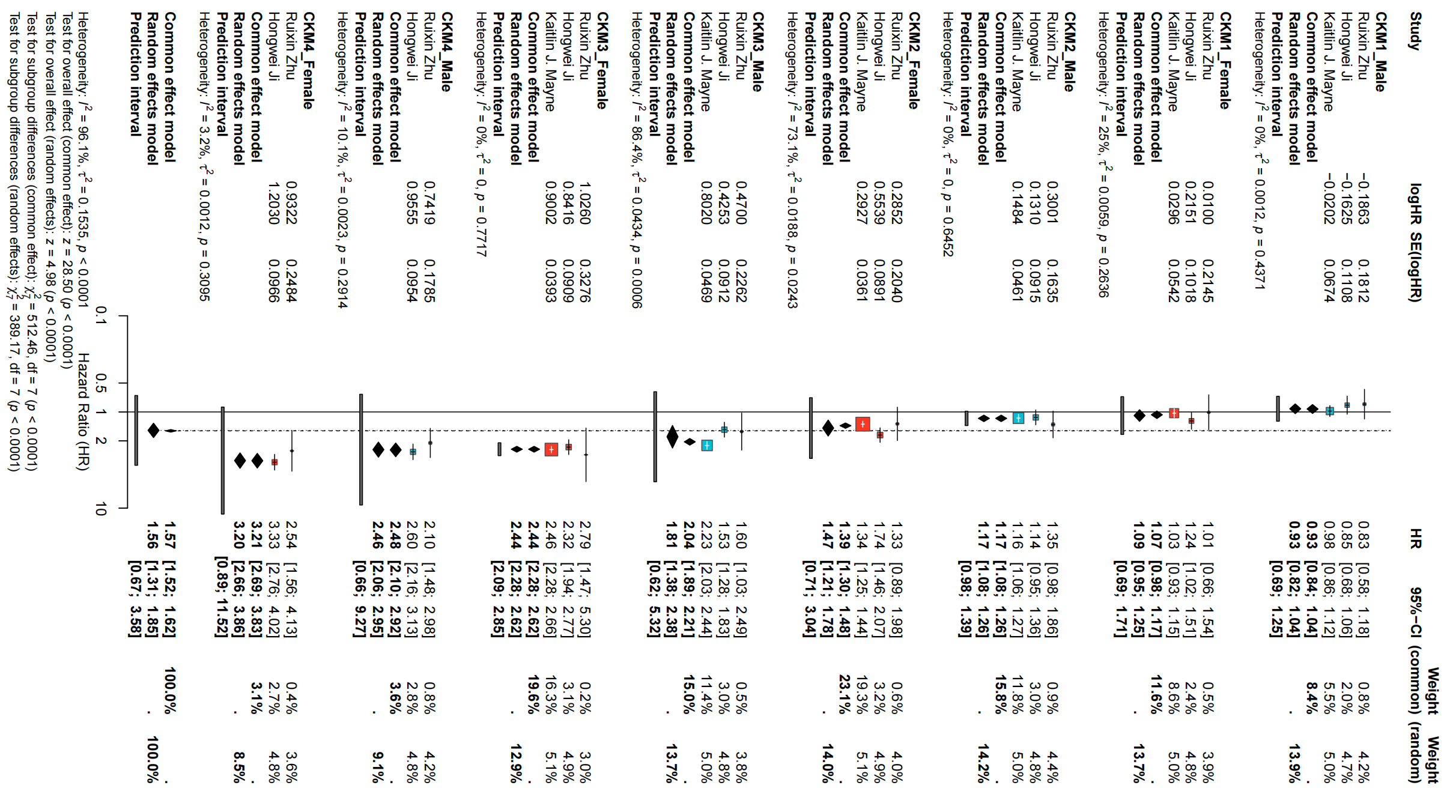


## Figure S2. Forest plot of CVD mortality by CKM stage and sex


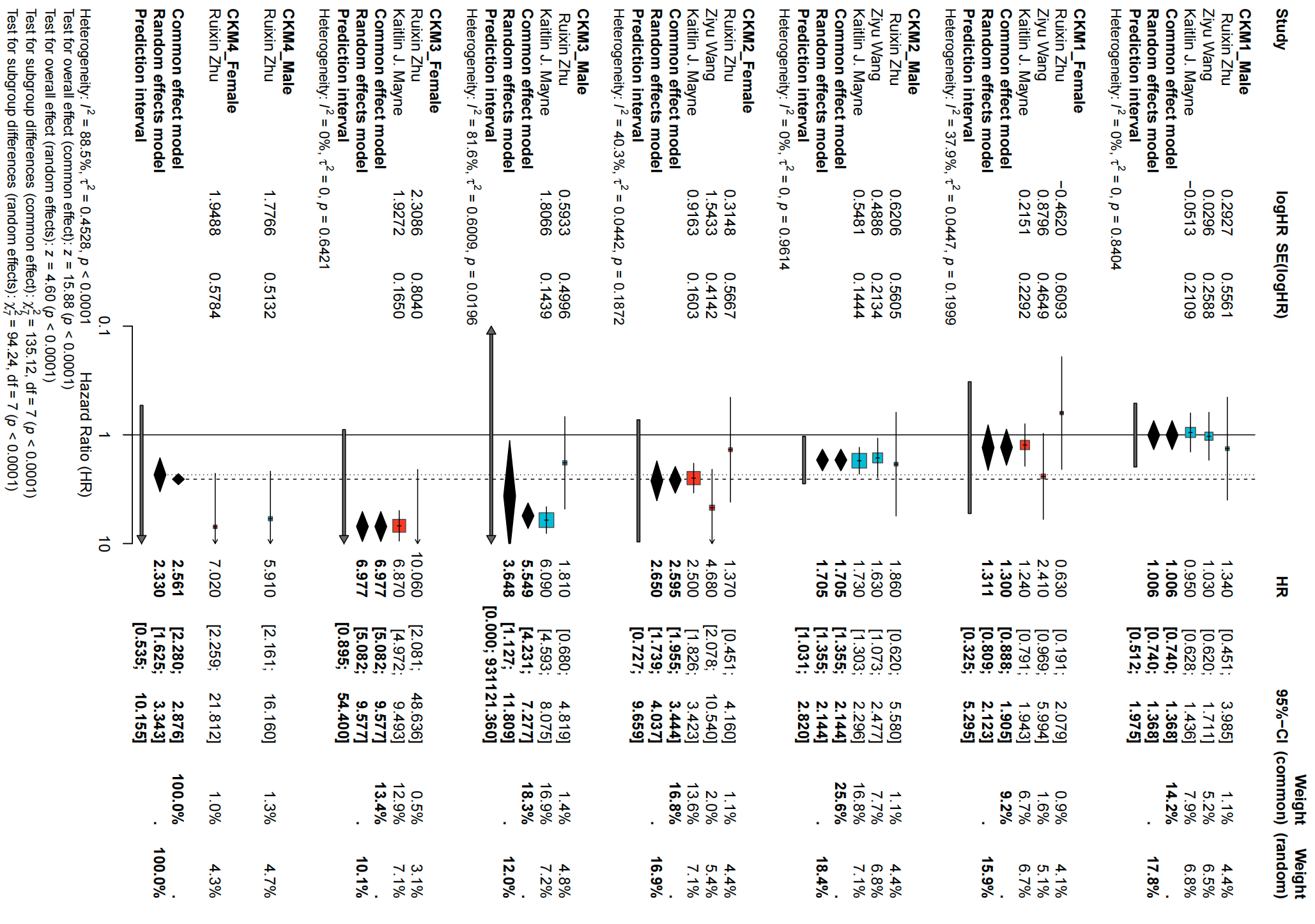


## Figure S3. Forest plot of CHD mortality by CKM stage


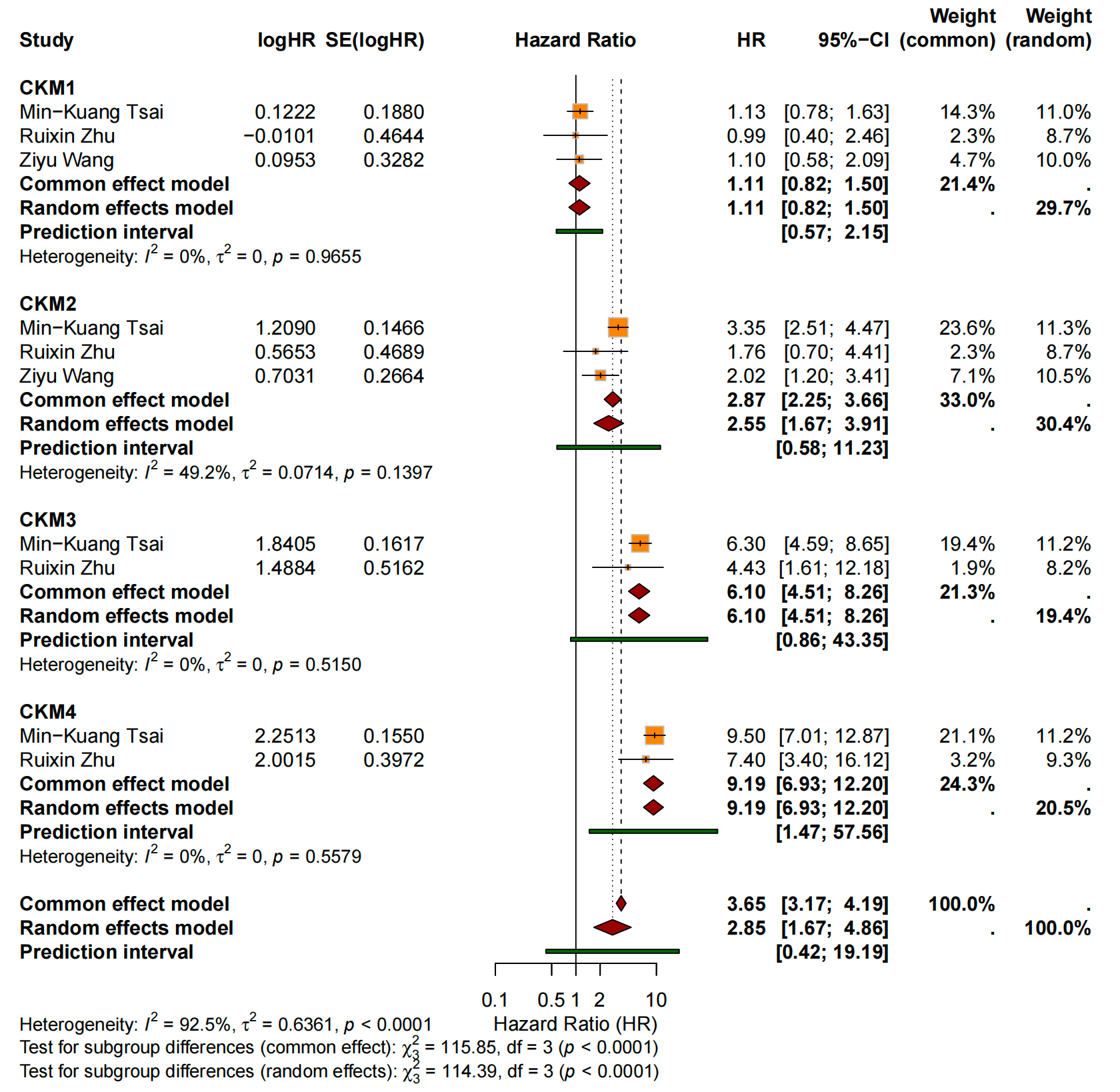


## Figure S4. Forest plot of stroke mortality by CKM stage


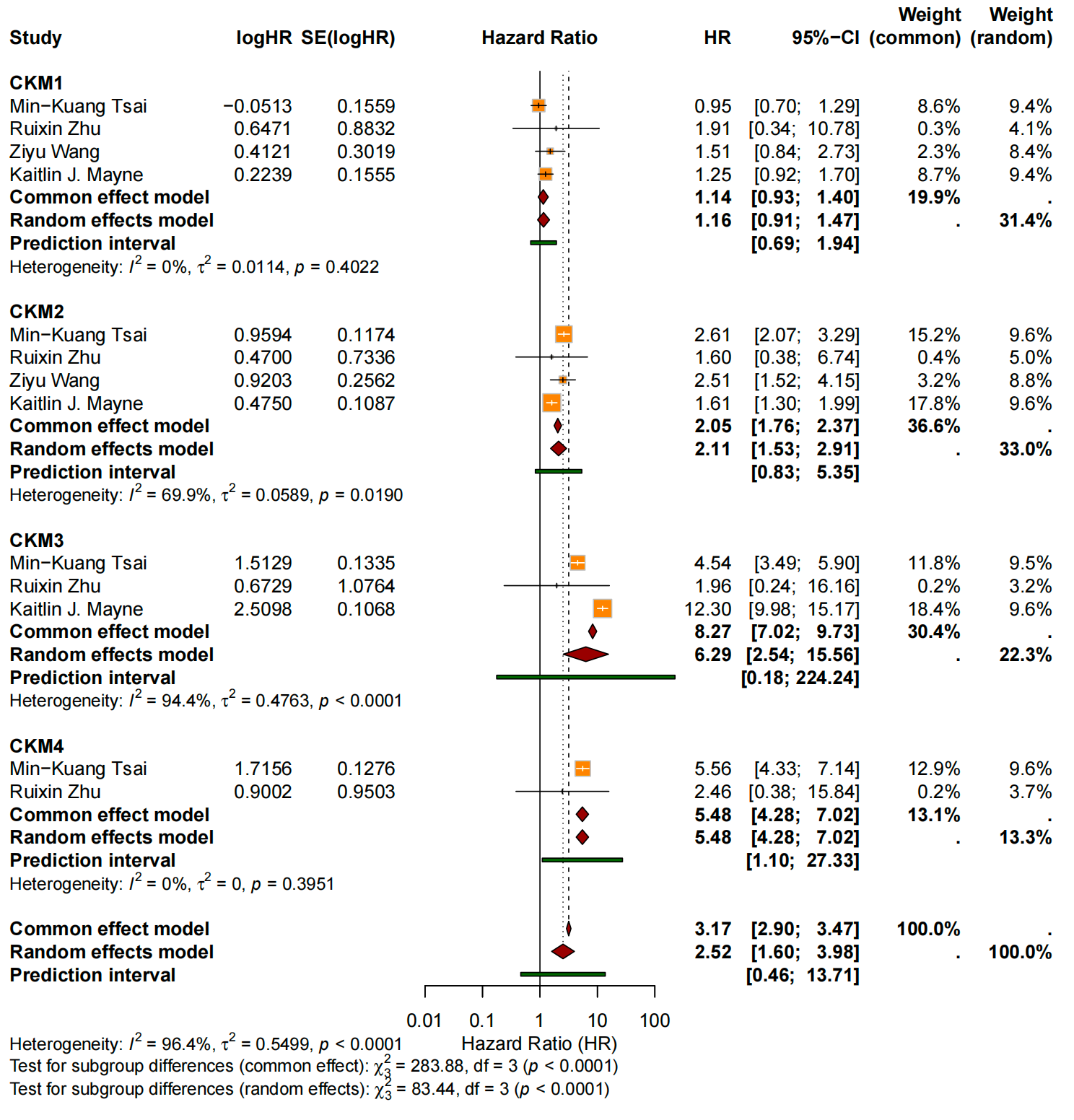


## Table S4. Meta-regression analyses of study-level moderators for all-cause, CVD, CHD, and stroke mortality

| Covariate | k | β (Estimate) | SE | 95% CI | t(df) | p-value | R² (%) | τ² | I² (%) | QE (p) |
| --- | --- | --- | --- | --- | --- | --- | --- | --- | --- | --- |
| *All-cause mortality* |  |  |  |  |  |  |  |  |  |  |
| Mean age | 23 | 0.0184 | 0.0179 | –0.0189 to 0.0556 | 1.02 (21) | 0.3175 | 0.09 | 0.5505 | 99.56 | 3862.01 (<0.001) |
| Female (%) | 23 | 0.0067 | 0.0076 | −0.0091 to 0.0225 | 0.88 (21) | 0.387 | 0 | 0.5561 | 99.6 | 4050.6 (<0.001) |
| Male (%) | 23 | −0.0067 | 0.0076 | −0.0225 to 0.0091 | −0.88 (21) | 0.387 | 0 | 0.5561 | 99.6 | 4050.6 (<0.001) |
| Sample size (log-scaled) | 23 | –0.1954 | 0.1053 | –0.4144 to 0.0235 | –1.86 (21) | 0.0775 | 10.69 | 0.4921 | 99.52 | 2872.83 (<0.001) |
| Mean BMI | 12 | 0.1333 | 0.2184 | –0.3533 to 0.6199 | 0.61 (10) | 0.5553 | 0 | 0.2531 | 99.22 | 630.51 (<0.001) |
| Physical inactivity (%) | 12 | 0.0045 | 0.0083 | –0.0139 to 0.0230 | 0.55 (10) | 0.5952 | 0 | 0.189 | 98.95 | 865.54 (<0.001) |
| Smoker (%) | 16 | 0.0065 | 0.0081 | –0.0108 to 0.0238 | 0.80 (14) | 0.4356 | 0 | 0.2225 | 98.75 | 881.12 (<0.001) |
| Drinker (%) | 16 | −0.0009 | 0.0054 | −0.0125 to 0.0108 | −0.16 (14) | 0.877 | 0 | 0.2335 | 98.9 | 821.6 (<0.001) |
| **Education < high school (%)** | **16** | **0.0083** | **0.0038** | **0.0001 to 0.0165** | **2.18 (14)** | **0.047** | **24.5** | **0.1639** | **98.2** | **329.2 (<0.001)** |
| Calendar period | 23 | 0 | 0 | −0.0000 to 0.0000 | 0.13 (21) | 0.901 | 0 | 0.5773 | 99.6 | 4257.8 (<0.001) |
| Follow-up years | 23 | −0.0004 | 0.0233 | −0.0488 to 0.0480 | −0.02 (21) | 0.986 | 0 | 0.5775 | 99.6 | 4051.6 (<0.001) |
| *CVD mortality* |  |  |  |  |  |  |  |  |  |  |
| **Mean age** | **14** | **0.0383** | **0.0172** | **0.0008 to 0.0759** | **2.22 (12)** | **0.046** | **22.47** | **0.3609** | **93.93** | **232.63 (<0.001)** |
| Female (%) | 14 | −0.0460 | 0.0223 | −0.0945 to 0.0025 | −2.07 (12) | 0.061 | 17.83 | 0.3825 | 94.28 | 357.94 (<0.001) |
| Male (%) | 14 | 0.046 | 0.0223 | −0.0025 to 0.0945 | 2.07 (12) | 0.061 | 17.83 | 0.3825 | 94.28 | 357.94 (<0.001) |
| Sample size | 18 | −0.0586 | 0.1186 | −0.3100 to 0.1928 | −0.49 (16) | 0.628 | 0 | 0.4686 | 96.15 | 432.02 (<0.001) |
| Mean BMI | 10 | −0.3484 | 0.4021 | −1.2757 to 0.5789 | −0.87 (8) | 0.412 | 0 | 0.532 | 97.48 | 358.05 (<0.001) |
| Physical inactivity (%) | 10 | −0.0166 | 0.0197 | −0.0619 to 0.0287 | −0.84 (8) | 0.423 | 0 | 0.4653 | 96.72 | 240.33 (<0.001) |
| Smoker (%) | 14 | 0.0132 | 0.0126 | −0.0142 to 0.0406 | 1.05 (12) | 0.315 | 0.32 | 0.464 | 95.64 | 364.86 (<0.001) |
| Drinker (%) | 14 | 0.0006 | 0.0083 | −0.0175 to 0.0187 | 0.08 (12) | 0.941 | 0 | 0.5037 | 96.28 | 363.87 (<0.001) |
| Education < high school (%) | 12 | 0.0031 | 0.0083 | −0.0153 to 0.0216 | 0.38 (10) | 0.714 | 0 | 0.5657 | 96.49 | 238.39 (<0.001) |
| Calendar period | 18 | −0.0049 | 0.0217 | −0.0508 to 0.0411 | −0.23 (16) | 0.825 | 0 | 0.4745 | 96.33 | 475.76 (<0.001) |
| Follow-up years | 18 | 0.0205 | 0.0209 | −0.0238 to 0.0649 | 0.98 (16) | 0.342 | 0 | 0.4473 | 96.1 | 459.29 (<0.001) |
| *CHD mortality* |  |  |  |  |  |  |  |  |  |  |
| Mean age | 10 | 0.0406 | 0.0236 | −0.0138 to 0.0950 | 1.72 (8) | 0.124 | 17.79 | 0.523 | 89.48 | 83.19 (<0.001) |
| Female (%) | 10 | −0.0506 | 0.0342 | −0.1296 to 0.0283 | −1.48 (8) | 0.177 | 8.6 | 0.5814 | 90.39 | 114.89 (<0.001) |
| Male (%) | 10 | 0.0506 | 0.0342 | −0.0283 to 0.1296 | 1.48 (8) | 0.177 | 8.6 | 0.5814 | 90.39 | 114.89 (<0.001) |
| Sample size | 10 | 0.0026 | 0.1629 | −0.3731 to 0.3783 | 0.02 (8) | 0.988 | 0 | 0.7279 | 92.53 | 118.69 (<0.001) |
| Mean BMI | 6 | 0.0656 | 1.0535 | −2.8595 to 2.9907 | 0.06 (4) | 0.953 | 0 | 0.9475 | 96.67 | 104.22 (<0.001) |
| Physical inactivity (%) | 10 | −0.0229 | 0.023 | −0.0760 to 0.0302 | −0.99 (8) | 0.35 | 3.1 | 0.6164 | 91.79 | 87.92 (<0.001) |
| Smoker (%) | 10 | 0.0124 | 0.0158 | −0.0240 to 0.0487 | 0.79 (8) | 0.455 | 0 | 0.6708 | 92.61 | 118.73 (<0.001) |
| Drinker (%) | 10 | −0.0005 | 0.0098 | −0.0232 to 0.0221 | −0.05 (8) | 0.958 | 0 | 0.72 | 93.29 | 120.59 (<0.001) |
| **Education < high school (%)** | **8** | **0.031** | **0.0114** | **0.0030 to 0.0589** | **2.71 (6)** | **0.035** | **54.82** | **0.2992** | **83.93** | **35.53 (<0.001)** |
| Calendar period | 10 | 0.0781 | 0.1096 | −0.1746 to 0.3308 | 0.71 (8) | 0.496 | 0 | 0.6698 | 92.82 | 112.72 (<0.001) |
| Follow-up years | 10 | 0.029 | 0.0361 | −0.0543 to 0.1122 | 0.80 (8) | 0.445 | 0 | 0.6598 | 92.53 | 107.61 (<0.001) |
| *Stroke mortality* |  |  |  |  |  |  |  |  |  |  |
| Mean age | 13 | 0.0301 | 0.0229 | −0.0202 to 0.0805 | 1.32 (11) | 0.214 | 5.67 | 0.5187 | 94.4 | 285.03 (<0.001) |
| Female (%) | 13 | −0.0115 | 0.0206 | −0.0569 to 0.0339 | −0.56 (11) | 0.588 | 0 | 0.5883 | 95.01 | 331.91 (<0.001) |
| Male (%) | 13 | 0.0115 | 0.0206 | −0.0339 to 0.0569 | 0.56 (11) | 0.588 | 0 | 0.5883 | 95.01 | 331.91 (<0.001) |
| Sample size | 13 | 0.0151 | 0.1186 | −0.2459 to 0.2761 | 0.13 (11) | 0.901 | 0 | 0.6015 | 95.2 | 319.62 (<0.001) |
| Mean BMI | 6 | 0.4653 | 0.7591 | −1.6423 to 2.5730 | 0.61 (4) | 0.573 | 0 | 0.4758 | 95.46 | 77.43 (<0.001) |
| Physical inactivity (%) | 10 | −0.0190 | 0.0148 | −0.0531 to 0.0152 | −1.28 (8) | 0.236 | 3.95 | 0.3189 | 87.99 | 74.84 (<0.001) |
| Smoker (%) | 10 | −0.0005 | 0.0142 | −0.0333 to 0.0323 | −0.04 (8) | 0.97 | 0 | 0.36 | 88.55 | 93.82 (<0.001) |
| Drinkers (%) | 10 | −0.0022 | 0.0083 | −0.0214 to 0.0169 | −0.27 (8) | 0.796 | 0 | 0.3594 | 89.74 | 89.66 (<0.001) |
| **Education < high school (%)** | **8** | **0.0271** | **0.0062** | **0.0120 to 0.0423** | **4.39 (6)** | **0.0046** | **67.61** | **0.1324** | **73.35** | **23.51 (0.0006)** |
| Calendar period | 13 | 0.0174 | 0.0395 | −0.0696 to 0.1043 | 0.44 (11) | 0.669 | 0 | 0.5984 | 94.98 | 327.77 (<0.001) |
| Follow-up years | 13 | 0.0177 | 0.0332 | −0.0555 to 0.0908 | 0.53 (11) | 0.606 | 0 | 0.5899 | 94.93 | 332.87 (<0.001) |

## Table S5. Subgroup meta-analysis for covariates showing significant differences in meta-regression (education level and mean age)

| **Model** | **k** | **logHR** | **logHR_se** | **tau2** | **Q** | **df_Q** | **HR** | **LCL** | **UCL** | **ed_median** |
| --- | --- | --- | --- | --- | --- | --- | --- | --- | --- | --- |
| *All-cause mortality* |  |  |  |  |  |  |  |  |  |  |
| High education group(n=8) | 8 | 0.433 | 0.145 | 0.155 | 722.647 | 7 | 1.542 | 1.161 | 2.048 | 71.55 |
| Low education group(n=8) | 8 | 0.683 | 0.174 | 0.227 | 166.230 | 7 | 1.979 | 1.407 | 2.783 | 71.55 |
| *CHD mortality* |  |  |  |  |  |  |  |  |  |  |
| High education group(n=8) | 4 | 0.659 | 0.458 | 0.689 | 19.316 | 3 | 1.934 | 0.788 | 4.746 | 25.1 |
| Low education group(n=8) | 4 | 1.724 | 0.275 | 0.243 | 24.618 | 3 | 5.606 | 3.268 | 9.617 | 25.1 |
| *Stroke mortality* |  |  |  |  |  |  |  |  |  |  |
| High education group(n=8) | 4 | 0.013 | 0.148 | 0.000 | 1.945 | 3 | 1.013 | 0.757 | 1.355 | 25.1 |
| Low education group(n=8) | 4 | 1.365 | 0.225 | 0.143 | 21.126 | 3 | 3.916 | 2.518 | 6.091 | 25.1 |
| *CVD mortality* |  |  |  |  |  |  |  |  |  |  |
| Age < median (n=7) | 7 | 0.581 | 0.351 | 0.786 | 301.601 | 6 | 1.789 | 0.899 | 3.559 | 57.55 |
| Age ≥ median (n=7) | 7 | 1.189 | 0.229 | 0.297 | 52.547 | 6 | 3.285 | 2.098 | 5.144 | 57.55 |

## Table S6. Leave-One-Out sensitivity analyses for covariates showing significant differences in meta-regression (education level and mean age)

|  | **Study_removed** | **logHR** | **logHR_se** | **tau2** | **Q** | **df_Q** | **HR** | **LCL** | **UCL** | **HR_baseline** | **Delta_logHR** | **Delta_HR_abs** |
| --- | --- | --- | --- | --- | --- | --- | --- | --- | --- | --- | --- | --- |
| **Education** |  |  |  |  |  |  |  |  |  |  |  |  |
| *All-cause mortality* | | | | | | | | | | | | |
|  | Hongwei Ji | 0.510 | 0.111 | 0.274 | 12273.794 | 22.000 | 1.665 | 1.340 | 2.070 | 1.931 | -0.148 | 0.265 |
|  | Haozhe Ding | 0.713 | 0.138 | 0.430 | 4271.085 | 22.000 | 2.040 | 1.555 | 2.675 | 1.931 | 0.055 | 0.109 |
|  | Ruixin Zhu | 0.701 | 0.120 | 0.323 | 14760.274 | 22.000 | 2.016 | 1.595 | 2.549 | 1.931 | 0.043 | 0.086 |
|  | Min-Kuang Tsai | 0.691 | 0.127 | 0.359 | 14024.751 | 22.000 | 1.995 | 1.556 | 2.558 | 1.931 | 0.033 | 0.065 |
|  | Kaitlin J. Mayne | 0.679 | 0.117 | 0.318 | 13453.305 | 23.000 | 1.972 | 1.568 | 2.479 | 1.931 | 0.021 | 0.041 |
|  | Na Li | 0.641 | 0.120 | 0.323 | 14584.300 | 22.000 | 1.898 | 1.500 | 2.402 | 1.931 | -0.017 | 0.033 |
|  | Congyi Zheng | 0.668 | 0.119 | 0.322 | 14700.949 | 22.000 | 1.949 | 1.543 | 2.464 | 1.931 | 0.010 | 0.019 |
| *CHD mortality* | | | | | | | | | | | | |
|  | Min-Kuang Tsai | 0.791 | 0.309 | 0.410 | 18.814 | 5.000 | 2.205 | 1.202 | 4.045 | 2.848 | -0.256 | 0.643 |
|  | Ziyu Wang | 1.211 | 0.313 | 0.675 | 100.180 | 7.000 | 3.357 | 1.818 | 6.202 | 2.848 | 0.164 | 0.509 |
|  | Ruixin Zhu | 1.059 | 0.357 | 0.718 | 106.913 | 5.000 | 2.883 | 1.432 | 5.804 | 2.848 | 0.012 | 0.034 |
| *Stroke mortality* | | | | | | | | | | | | |
|  | Min-Kuang Tsai | 0.830 | 0.428 | 1.341 | 243.896 | 8.000 | 2.293 | 0.992 | 5.301 | 2.496 | -0.085 | 0.203 |
|  | Ruixin Zhu | 0.972 | 0.303 | 0.798 | 331.802 | 8.000 | 2.644 | 1.459 | 4.791 | 2.496 | 0.058 | 0.148 |
|  | Ziyu Wang | 0.963 | 0.305 | 0.827 | 326.128 | 10.000 | 2.620 | 1.440 | 4.766 | 2.496 | 0.049 | 0.124 |
|  | Kaitlin J. Mayne | 0.873 | 0.246 | 0.415 | 95.348 | 9.000 | 2.394 | 1.480 | 3.874 | 2.496 | -0.042 | 0.102 |
| Age |  |  |  |  |  |  |  |  |  |  |  |  |
| *CVD mortality* | | | | | | | | | | | | |
|  | Congyi Zheng | 1.026 | 0.196 | 0.478 | 440.965 | 13.000 | 2.790 | 1.901 | 4.095 | 2.568 | 0.083 | 0.222 |
|  | Min-Kuang Tsai | 0.856 | 0.184 | 0.402 | 168.142 | 13.000 | 2.354 | 1.640 | 3.378 | 2.568 | -0.087 | 0.214 |
|  | Ziyu Wang | 0.994 | 0.185 | 0.486 | 463.650 | 15.000 | 2.701 | 1.880 | 3.880 | 2.568 | 0.051 | 0.133 |
|  | Mian Li | 0.895 | 0.211 | 0.547 | 371.100 | 13.000 | 2.447 | 1.620 | 3.697 | 2.568 | -0.048 | 0.120 |
|  | Ruixin Zhu | 0.933 | 0.191 | 0.482 | 472.735 | 13.000 | 2.542 | 1.749 | 3.696 | 2.568 | -0.010 | 0.025 |

## Table S7. All Outcomes Leave-One-Out Sensitivity Analysis

| Study_removed | logHR | logHR_se | tau2 | Q | df_Q | HR | LCL | UCL | HR_baseline | Delta_logHR | Delta_HR_abs |
| --- | --- | --- | --- | --- | --- | --- | --- | --- | --- | --- | --- |
| *All-cause mortality* |  |  |  |  |  |  |  |  |  |  |  |
| Congyi Zheng_1_1 | 0.690 | 0.113 | 0.322 | 14771.935 | 25 | 1.993 | 1.599 | 2.485 | 1.931 | 0.032 | 0.063 |
| Congyi Zheng_1_2 | 0.671 | 0.113 | 0.322 | 14781.301 | 25 | 1.956 | 1.568 | 2.440 | 1.931 | 0.013 | 0.025 |
| Congyi Zheng_1_3 | 0.649 | 0.113 | 0.322 | 14765.796 | 25 | 1.913 | 1.534 | 2.386 | 1.931 | -0.009 | 0.018 |
| Congyi Zheng_1_4 | 0.631 | 0.113 | 0.321 | 14726.779 | 25 | 1.879 | 1.507 | 2.343 | 1.931 | -0.027 | 0.052 |
| Min-Kuang Tsai_3_5 | 0.685 | 0.114 | 0.327 | 14563.988 | 25 | 1.984 | 1.588 | 2.480 | 1.931 | 0.028 | 0.054 |
| Min-Kuang Tsai_3_6 | 0.672 | 0.116 | 0.338 | 14769.651 | 25 | 1.957 | 1.561 | 2.455 | 1.931 | 0.014 | 0.027 |
| Min-Kuang Tsai_3_7 | 0.654 | 0.114 | 0.328 | 14594.121 | 25 | 1.923 | 1.538 | 2.404 | 1.931 | -0.004 | 0.008 |
| Min-Kuang Tsai_3_8 | 0.650 | 0.114 | 0.326 | 14428.971 | 25 | 1.915 | 1.533 | 2.392 | 1.931 | -0.008 | 0.016 |
| Ruixin Zhu_5_9 | 0.687 | 0.113 | 0.322 | 14769.328 | 25 | 1.988 | 1.594 | 2.479 | 1.931 | 0.029 | 0.057 |
| Ruixin Zhu_5_10 | 0.672 | 0.113 | 0.322 | 14780.978 | 25 | 1.959 | 1.570 | 2.443 | 1.931 | 0.014 | 0.028 |
| Ruixin Zhu_5_11 | 0.658 | 0.113 | 0.322 | 14779.678 | 25 | 1.930 | 1.548 | 2.407 | 1.931 | 0.000 | 0.000 |
| Ruixin Zhu_5_12 | 0.653 | 0.113 | 0.322 | 14775.132 | 25 | 1.922 | 1.541 | 2.397 | 1.931 | -0.005 | 0.009 |
| Hongwei Ji_8_13 | 0.679 | 0.113 | 0.323 | 14769.176 | 25 | 1.971 | 1.579 | 2.460 | 1.931 | 0.021 | 0.040 |
| Hongwei Ji_8_14 | 0.653 | 0.113 | 0.323 | 14744.447 | 25 | 1.922 | 1.540 | 2.399 | 1.931 | -0.004 | 0.008 |
| Hongwei Ji_8_15 | 0.587 | 0.109 | 0.297 | 13568.248 | 25 | 1.798 | 1.454 | 2.225 | 1.931 | -0.071 | 0.132 |
| Hongwei Ji_8_16 | 0.582 | 0.108 | 0.297 | 13550.549 | 25 | 1.790 | 1.447 | 2.214 | 1.931 | -0.076 | 0.141 |
| Haozhe Ding_19_17 | 0.692 | 0.105 | 0.280 | 9785.737 | 25 | 1.998 | 1.625 | 2.457 | 1.931 | 0.034 | 0.068 |
| Haozhe Ding_19_18 | 0.678 | 0.126 | 0.402 | 13796.981 | 25 | 1.969 | 1.539 | 2.520 | 1.931 | 0.020 | 0.038 |
| Haozhe Ding_19_19 | 0.669 | 0.125 | 0.398 | 14774.434 | 25 | 1.952 | 1.528 | 2.495 | 1.931 | 0.011 | 0.022 |
| Haozhe Ding_19_20 | 0.642 | 0.096 | 0.233 | 8264.479 | 25 | 1.901 | 1.573 | 2.297 | 1.931 | -0.015 | 0.030 |
| Na Li_20_21 | 0.675 | 0.113 | 0.323 | 14777.141 | 25 | 1.964 | 1.574 | 2.451 | 1.931 | 0.017 | 0.033 |
| Na Li_20_22 | 0.662 | 0.113 | 0.323 | 14777.358 | 25 | 1.939 | 1.554 | 2.420 | 1.931 | 0.004 | 0.009 |
| Na Li_20_23 | 0.647 | 0.113 | 0.322 | 14728.758 | 25 | 1.909 | 1.530 | 2.381 | 1.931 | -0.011 | 0.022 |
| Na Li_20_24 | 0.632 | 0.112 | 0.320 | 14646.380 | 25 | 1.882 | 1.510 | 2.346 | 1.931 | -0.026 | 0.049 |
| Kaitlin J. Mayne_22_25 | 0.680 | 0.114 | 0.327 | 14718.089 | 25 | 1.974 | 1.580 | 2.466 | 1.931 | 0.022 | 0.043 |
| Kaitlin J. Mayne_22_26 | 0.682 | 0.114 | 0.328 | 14630.719 | 25 | 1.979 | 1.583 | 2.474 | 1.931 | 0.025 | 0.048 |
| Kaitlin J. Mayne_22_27 | 0.631 | 0.110 | 0.306 | 13644.090 | 25 | 1.879 | 1.514 | 2.331 | 1.931 | -0.027 | 0.052 |
| *CVD mortality* |  |  |  |  |  |  |  |  |  |  |  |
| Congyi Zheng_1b_1 | 1.001 | 0.175 | 0.467 | 467.077 | 16 | 2.722 | 1.930 | 3.839 | 2.568 | 0.058 | 0.154 |
| Congyi Zheng_1c_2 | 0.991 | 0.176 | 0.468 | 462.466 | 16 | 2.693 | 1.908 | 3.803 | 2.568 | 0.048 | 0.126 |
| Congyi Zheng_1d_3 | 0.944 | 0.180 | 0.490 | 481.838 | 16 | 2.570 | 1.807 | 3.655 | 2.568 | 0.001 | 0.002 |
| Congyi Zheng_1e_4 | 0.904 | 0.179 | 0.487 | 479.180 | 16 | 2.470 | 1.738 | 3.509 | 2.568 | -0.039 | 0.098 |
| Min-Kuang Tsai_3b_5 | 1.002 | 0.159 | 0.371 | 331.687 | 16 | 2.723 | 1.994 | 3.720 | 2.568 | 0.059 | 0.156 |
| Min-Kuang Tsai_3c_6 | 0.933 | 0.195 | 0.585 | 480.757 | 16 | 2.542 | 1.734 | 3.726 | 2.568 | -0.010 | 0.026 |
| Min-Kuang Tsai_3d_7 | 0.895 | 0.184 | 0.511 | 436.806 | 16 | 2.448 | 1.708 | 3.509 | 2.568 | -0.048 | 0.120 |
| Min-Kuang Tsai_3e_8 | 0.875 | 0.163 | 0.393 | 334.163 | 16 | 2.399 | 1.742 | 3.304 | 2.568 | -0.068 | 0.169 |
| Ruixin Zhu_5b_9 | 0.981 | 0.176 | 0.476 | 477.775 | 16 | 2.667 | 1.888 | 3.767 | 2.568 | 0.038 | 0.099 |
| Ruixin Zhu_5c_10 | 0.961 | 0.177 | 0.480 | 481.377 | 16 | 2.613 | 1.848 | 3.695 | 2.568 | 0.018 | 0.046 |
| Ruixin Zhu_5d_11 | 0.924 | 0.177 | 0.481 | 482.954 | 16 | 2.520 | 1.782 | 3.564 | 2.568 | -0.019 | 0.048 |
| Ruixin Zhu_5e_12 | 0.898 | 0.177 | 0.479 | 480.006 | 16 | 2.454 | 1.735 | 3.471 | 2.568 | -0.045 | 0.114 |
| Mian Li_10b_13 | 0.994 | 0.171 | 0.437 | 409.816 | 16 | 2.701 | 1.931 | 3.778 | 2.568 | 0.051 | 0.133 |
| Mian Li_10c_14 | 0.945 | 0.185 | 0.518 | 476.947 | 16 | 2.573 | 1.792 | 3.695 | 2.568 | 0.002 | 0.005 |
| Mian Li_10d_15 | 0.907 | 0.184 | 0.510 | 474.727 | 16 | 2.477 | 1.729 | 3.549 | 2.568 | -0.036 | 0.091 |
| Mian Li_10e_16 | 0.889 | 0.180 | 0.486 | 451.584 | 16 | 2.432 | 1.711 | 3.457 | 2.568 | -0.054 | 0.136 |
| Ziyu Wang_17b_17 | 0.983 | 0.177 | 0.473 | 467.363 | 16 | 2.673 | 1.890 | 3.781 | 2.568 | 0.040 | 0.106 |
| Ziyu Wang_17c_18 | 0.950 | 0.180 | 0.491 | 479.756 | 16 | 2.586 | 1.817 | 3.681 | 2.568 | 0.007 | 0.019 |
| *CHD mortality* |  |  |  |  |  |  |  |  |  |  |  |
| Min-Kuang Tsai_3g_1 | 1.171 | 0.260 | 0.498 | 75.259 | 8 | 3.226 | 1.938 | 5.371 | 2.848 | 0.125 | 0.378 |
| Min-Kuang Tsai_3h_2 | 1.021 | 0.340 | 0.921 | 120.183 | 8 | 2.777 | 1.427 | 5.404 | 2.848 | -0.025 | 0.071 |
| Min-Kuang Tsai_3i_3 | 0.946 | 0.315 | 0.774 | 106.463 | 8 | 2.576 | 1.391 | 4.771 | 2.848 | -0.101 | 0.273 |
| Min-Kuang Tsai_3j_4 | 0.897 | 0.264 | 0.515 | 72.313 | 8 | 2.453 | 1.462 | 4.117 | 2.848 | -0.149 | 0.395 |
| Ruixin Zhu_5g_5 | 1.149 | 0.289 | 0.658 | 112.548 | 8 | 3.153 | 1.789 | 5.558 | 2.848 | 0.102 | 0.305 |
| Ruixin Zhu_5h_6 | 1.093 | 0.296 | 0.693 | 118.153 | 8 | 2.982 | 1.670 | 5.323 | 2.848 | 0.046 | 0.133 |
| Ruixin Zhu_5i_7 | 1.006 | 0.297 | 0.704 | 120.482 | 8 | 2.734 | 1.528 | 4.895 | 2.848 | -0.041 | 0.114 |
| Ruixin Zhu_5j_8 | 0.947 | 0.298 | 0.697 | 117.351 | 8 | 2.579 | 1.440 | 4.620 | 2.848 | -0.099 | 0.269 |
| Ziyu Wang_17e_9 | 1.153 | 0.288 | 0.642 | 106.623 | 8 | 3.168 | 1.802 | 5.570 | 2.848 | 0.106 | 0.320 |
| Ziyu Wang_17f_10 | 1.086 | 0.304 | 0.722 | 115.323 | 8 | 2.961 | 1.632 | 5.373 | 2.848 | 0.039 | 0.113 |
| *Stroke mortality* |  |  |  |  |  |  |  |  |  |  |  |
| Min-Kuang Tsai_3l_1 | 1.016 | 0.271 | 0.698 | 268.001 | 11 | 2.762 | 1.624 | 4.698 | 2.496 | 0.102 | 0.267 |
| Min-Kuang Tsai_3m_2 | 0.905 | 0.308 | 0.938 | 330.071 | 11 | 2.472 | 1.351 | 4.522 | 2.496 | -0.010 | 0.024 |
| Min-Kuang Tsai_3n_3 | 0.851 | 0.301 | 0.887 | 325.047 | 11 | 2.342 | 1.299 | 4.223 | 2.496 | -0.063 | 0.153 |
| Min-Kuang Tsai_3o_4 | 0.831 | 0.296 | 0.858 | 310.964 | 11 | 2.297 | 1.285 | 4.106 | 2.496 | -0.083 | 0.199 |
| Ruixin Zhu_5l_5 | 0.928 | 0.278 | 0.785 | 332.953 | 11 | 2.529 | 1.465 | 4.364 | 2.496 | 0.013 | 0.033 |
| Ruixin Zhu_5m_6 | 0.941 | 0.280 | 0.785 | 332.412 | 11 | 2.562 | 1.481 | 4.432 | 2.496 | 0.026 | 0.066 |
| Ruixin Zhu_5n_7 | 0.924 | 0.277 | 0.785 | 333.083 | 11 | 2.519 | 1.464 | 4.336 | 2.496 | 0.009 | 0.024 |
| Ruixin Zhu_5o_8 | 0.915 | 0.278 | 0.786 | 333.211 | 11 | 2.497 | 1.448 | 4.305 | 2.496 | 0.001 | 0.001 |
| Ziyu Wang_17h_9 | 0.961 | 0.285 | 0.792 | 327.114 | 11 | 2.613 | 1.495 | 4.568 | 2.496 | 0.046 | 0.117 |
| Ziyu Wang_17i_10 | 0.913 | 0.289 | 0.815 | 332.429 | 11 | 2.491 | 1.414 | 4.390 | 2.496 | -0.002 | 0.004 |
| Kaitlin J. Mayne_22b_11 | 0.985 | 0.283 | 0.770 | 294.173 | 11 | 2.677 | 1.538 | 4.657 | 2.496 | 0.070 | 0.181 |
| Kaitlin J. Mayne_22c_12 | 0.957 | 0.292 | 0.830 | 286.000 | 11 | 2.605 | 1.469 | 4.620 | 2.496 | 0.043 | 0.109 |
| Kaitlin J. Mayne_22d_13 | 0.761 | 0.209 | 0.379 | 135.585 | 11 | 2.139 | 1.420 | 3.223 | 2.496 | -0.154 | 0.357 |

Leave-one-out sensitivity analyses were performed for all four mortality outcomes (all-cause, cardiovascular disease [CVD], coronary heart disease [CHD], and stroke mortality) to assess the robustness of pooled hazard ratios (HRs) and identify influential studies contributing to heterogeneity. For each outcome, sequential exclusion of individual studies yielded highly consistent estimates: the recalculated logHRs fluctuated within ±0.03 of the baseline value and all pooled HRs remained statistically significant in the same direction as the main analysis.

All-cause mortality: Excluding any single study produced HRs between 1.88 and 1.99 (95% CI ≈ 1.51–2.48), confirming that the overall estimate (HR = 1.93) was stable. Although Min-Kuang Tsai (3_7) and Congyi Zheng (1_5) showed slightly greater influence and heterogeneity contribution, their removal did not materially change the effect direction or precision.

CVD mortality: Re-estimation after removing each study produced HRs ranging 2.40–2.72 with ΔHR < 0.16; Min-Kuang Tsai (3b_7) and Mian Li (10b_13) contributed modestly higher heterogeneity (Het_contribution > 100), but pooled results were essentially unchanged.

CHD mortality: Sensitivity results were consistent (HR ≈ 2.45–3.23). While Min-Kuang Tsai (3j_4) and Ruixin Zhu (5g_5) had slightly larger influence values (ΔHR ≈ 0.30–0.38), excluding them did not affect overall significance, indicating the robustness of CHD mortality findings.

Stroke mortality: Excluding individual trials changed HRs within ±0.15, with Kaitlin J. Mayne (22d_13) showing the largest deviation (ΔHR = 0.36). Nonetheless, pooled HRs remained > 2.0 and statistically significant, supporting a stable relationship between advanced CKM stages and stroke mortality.

## Figure S5. Baujat plot


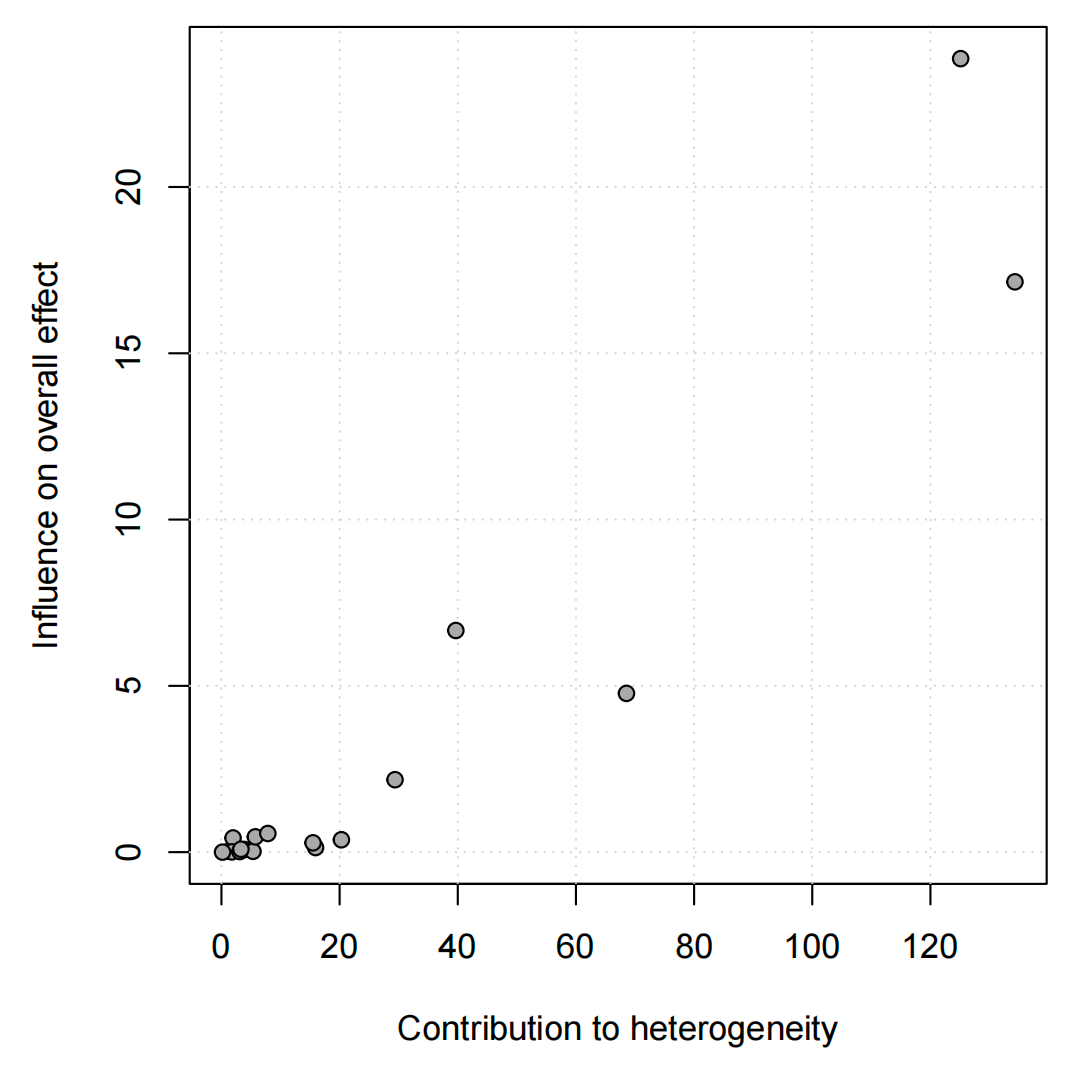
*All-cause mortality CVD mortality*


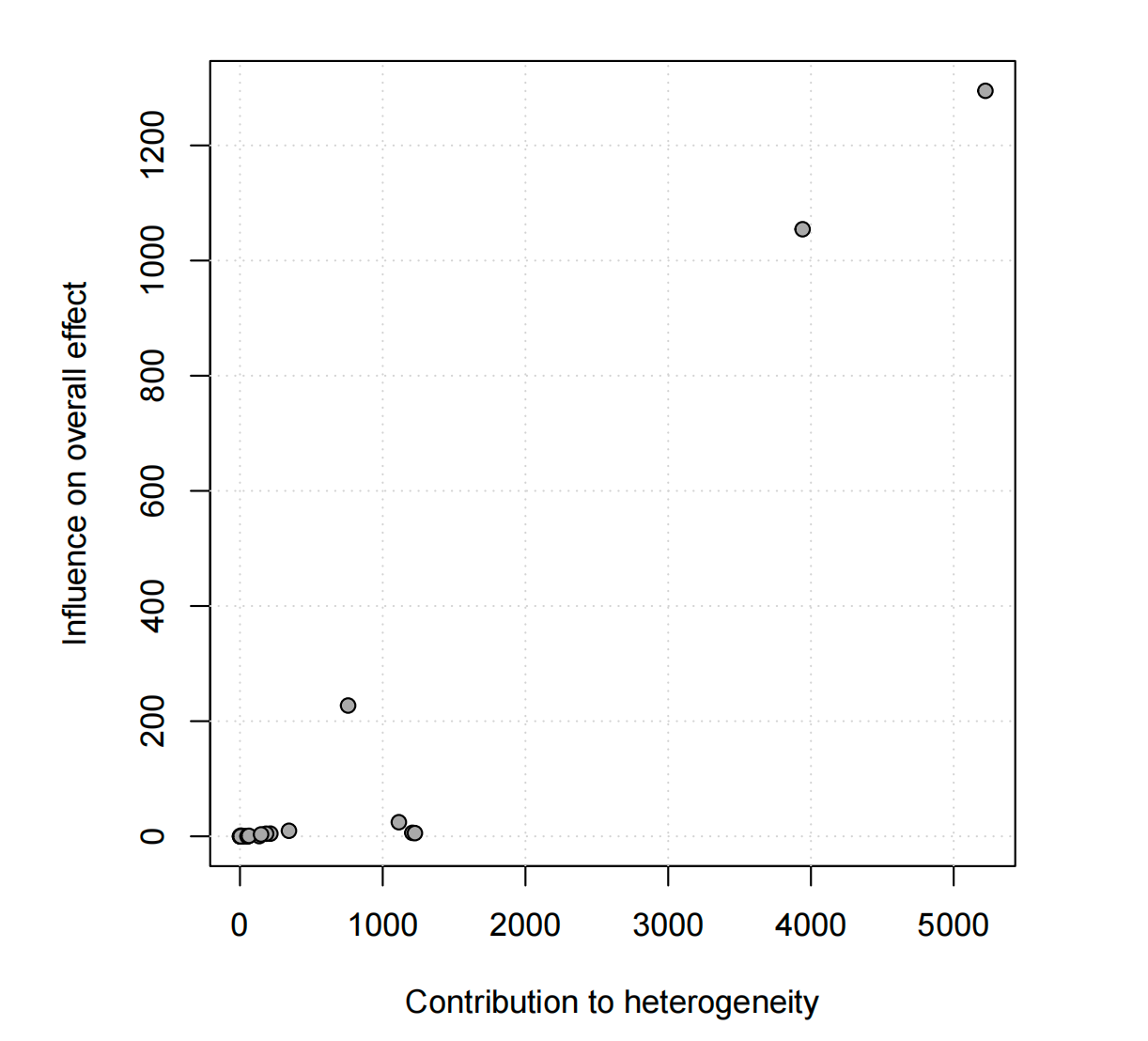


*CHD mortality Stroke mortality*


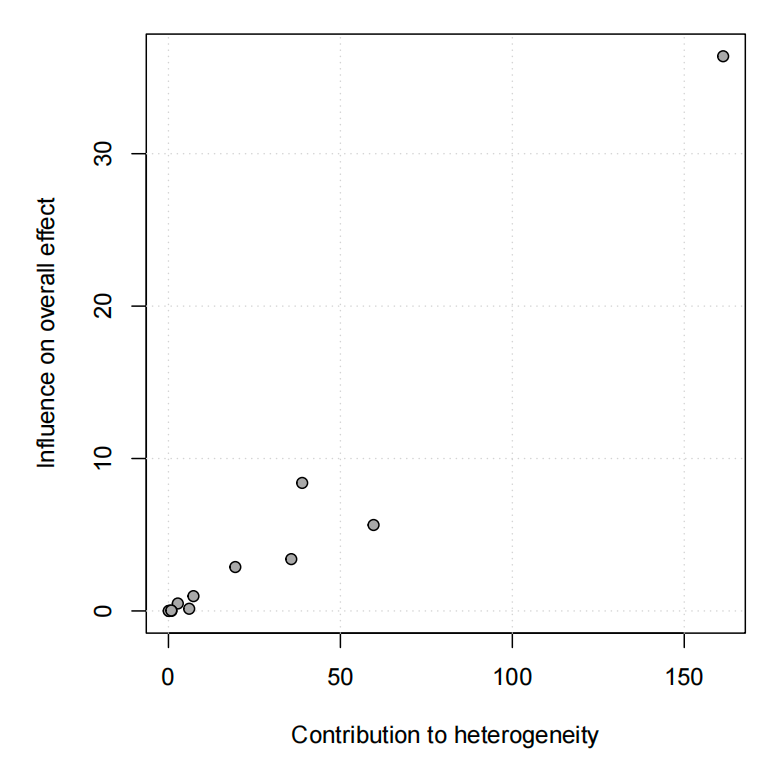

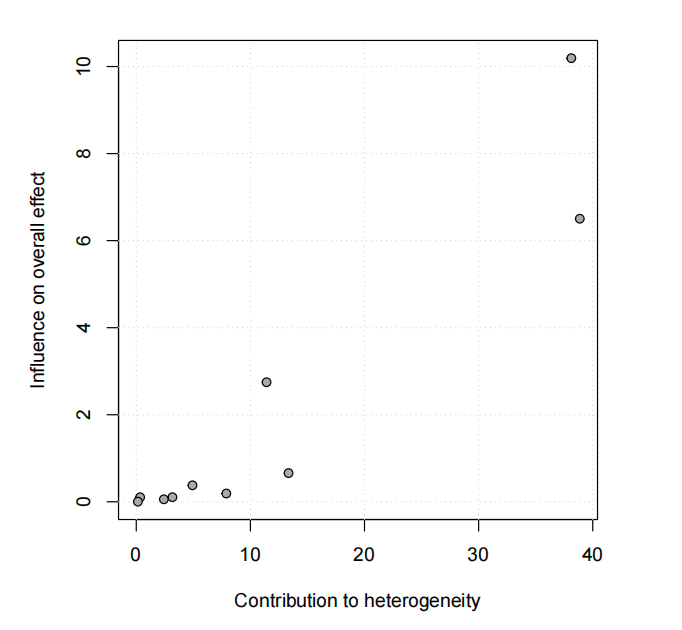


## Table S8. Baujat Sensitivity Analysis Across All Outcomes

| **Study** | **Het_contribution** | **Influence_on_overall** |
| --- | --- | --- |
| *All-cause mortality* |  |  |
| Congyi Zheng_1_2 | Congyi Zheng_1_2 | 9.680 |
| Congyi Zheng_1_3 | Congyi Zheng_1_3 | 0.318 |
| Congyi Zheng_1_4 | Congyi Zheng_1_4 | 15.807 |
| Congyi Zheng_1_5 | Congyi Zheng_1_5 | 54.786 |
| Min-Kuang Tsai_3_7 | Min-Kuang Tsai_3_7 | 212.966 |
| Min-Kuang Tsai_3_8 | Min-Kuang Tsai_3_8 | 11.540 |
| Min-Kuang Tsai_3_9 | Min-Kuang Tsai_3_9 | 182.998 |
| Min-Kuang Tsai_3_10 | Min-Kuang Tsai_3_10 | 343.066 |
| Ruixin Zhu_5_12 | Ruixin Zhu_5_12 | 12.281 |
| Ruixin Zhu_5_13 | Ruixin Zhu_5_13 | 0.641 |
| Ruixin Zhu_5_14 | Ruixin Zhu_5_14 | 1.940 |
| Ruixin Zhu_5_15 | Ruixin Zhu_5_15 | 6.483 |
| Hongwei Ji_8_17 | Hongwei Ji_8_17 | 12.402 |
| Hongwei Ji_8_18 | Hongwei Ji_8_18 | 37.004 |
| Hongwei Ji_8_19 | Hongwei Ji_8_19 | 1207.295 |
| Hongwei Ji_8_20 | Hongwei Ji_8_20 | 1225.507 |
| Haozhe Ding_19_22 | Haozhe Ding_19_22 | 3941.471 |
| Haozhe Ding_19_23 | Haozhe Ding_19_23 | 757.498 |
| Haozhe Ding_19_24 | Haozhe Ding_19_24 | 6.047 |
| Haozhe Ding_19_25 | Haozhe Ding_19_25 | 5222.305 |
| Na Li_20_27 | Na Li_20_27 | 4.465 |
| Na Li_20_28 | Na Li_20_28 | 4.249 |
| Na Li_20_29 | Na Li_20_29 | 52.702 |
| Na Li_20_30 | Na Li_20_30 | 134.858 |
| Kaitlin J. Mayne_22_32 | Kaitlin J. Mayne_22_32 | 62.657 |
| Kaitlin J. Mayne_22_33 | Kaitlin J. Mayne_22_33 | 147.608 |
| Kaitlin J. Mayne_22_34 | Kaitlin J. Mayne_22_34 | 1113.047 |
| *CVD mortality* |  |  |
| Congyi Zheng_1b_2 | Congyi Zheng_1b_2 | 15.926 |
| Congyi Zheng_1c_3 | Congyi Zheng_1c_3 | 20.302 |
| Congyi Zheng_1d_4 | Congyi Zheng_1d_4 | 1.275 |
| Congyi Zheng_1e_5 | Congyi Zheng_1e_5 | 3.881 |
| Min-Kuang Tsai_3b_7 | Min-Kuang Tsai_3b_7 | 134.305 |
| Min-Kuang Tsai_3c_8 | Min-Kuang Tsai_3c_8 | 1.958 |
| Min-Kuang Tsai_3d_9 | Min-Kuang Tsai_3d_9 | 39.670 |
| Min-Kuang Tsai_3e_10 | Min-Kuang Tsai_3e_10 | 125.118 |
| Ruixin Zhu_5b_22 | Ruixin Zhu_5b_22 | 5.341 |
| Ruixin Zhu_5c_23 | Ruixin Zhu_5c_23 | 1.756 |
| Ruixin Zhu_5d_24 | Ruixin Zhu_5d_24 | 0.185 |
| Ruixin Zhu_5e_25 | Ruixin Zhu_5e_25 | 3.116 |
| Mian Li_10b_37 | Mian Li_10b_37 | 68.550 |
| Mian Li_10c_38 | Mian Li_10c_38 | 5.733 |
| Mian Li_10d_39 | Mian Li_10d_39 | 7.852 |
| Mian Li_10e_40 | Mian Li_10e_40 | 29.378 |
| Ziyu Wang_17b_42 | Ziyu Wang_17b_42 | 15.497 |
| Ziyu Wang_17c_43 | Ziyu Wang_17c_43 | 3.298 |
| *CHD mortality* |  |  |
| Min-Kuang Tsai_3g_12 | Min-Kuang Tsai_3g_12 | 38.864 |
| Min-Kuang Tsai_3h_13 | Min-Kuang Tsai_3h_13 | 0.339 |
| Min-Kuang Tsai_3i_14 | Min-Kuang Tsai_3i_14 | 11.417 |
| Min-Kuang Tsai_3j_15 | Min-Kuang Tsai_3j_15 | 38.121 |
| Ruixin Zhu_5g_27 | Ruixin Zhu_5g_27 | 7.889 |
| Ruixin Zhu_5h_28 | Ruixin Zhu_5h_28 | 2.417 |
| Ruixin Zhu_5i_29 | Ruixin Zhu_5i_29 | 0.141 |
| Ruixin Zhu_5j_30 | Ruixin Zhu_5j_30 | 3.170 |
| Ziyu Wang_17e_45 | Ziyu Wang_17e_45 | 13.345 |
| Ziyu Wang_17f_46 | Ziyu Wang_17f_46 | 4.925 |
| *Stroke mortality* |  |  |
| Min-Kuang Tsai_3l_17 | Min-Kuang Tsai_3l_17 | 59.642 |
| Min-Kuang Tsai_3m_18 | Min-Kuang Tsai_3m_18 | 2.722 |
| Min-Kuang Tsai_3n_19 | Min-Kuang Tsai_3n_19 | 7.265 |
| Min-Kuang Tsai_3o_20 | Min-Kuang Tsai_3o_20 | 19.439 |
| Ruixin Zhu_5l_32 | Ruixin Zhu_5l_32 | 0.328 |
| Ruixin Zhu_5m_33 | Ruixin Zhu_5m_33 | 0.867 |
| Ruixin Zhu_5n_34 | Ruixin Zhu_5n_34 | 0.199 |
| Ruixin Zhu_5o_35 | Ruixin Zhu_5o_35 | 0.071 |
| Ziyu Wang_17h_48 | Ziyu Wang_17h_48 | 6.025 |
| Ziyu Wang_17i_49 | Ziyu Wang_17i_49 | 0.825 |
| Kaitlin J. Mayne_22b_51 | Kaitlin J. Mayne_22b_51 | 35.711 |
| Kaitlin J. Mayne_22c_52 | Kaitlin J. Mayne_22c_52 | 38.883 |
| Kaitlin J. Mayne_22d_53 | Kaitlin J. Mayne_22d_53 | 161.304 |

The accompanying Baujat analysis (Table S7 and Figure S6) identified studies with relatively higher contributions to between-study heterogeneity, such as Haozhe Ding (19_25) in all-cause mortality and Kaitlin J. Mayne (22d_53) in stroke mortality; however, none exerted a disproportionate impact on the summary estimates.

Interpretation: Across all outcomes, both leave-one-out and Baujat analyses demonstrated that the pooled results were not driven by any single study. These findings confirm the high stability and internal consistency of the meta-analytic conclusions.

## Table S9. Methodological quality of included cohort studies based on the JBI Critical Appraisal Checklist

| **Study** | **1. Similar groups & same population?** | **2. Exposures measured similarly?** | **3. Exposure measured validly/reliably?** | **4.Confounders identified?** | **5. Strategy for confounding?** | **6.Outcome free at start?** | **7. Outcome measured validly/reliably?** | **8.Follow-up sufficient & reported?** | **9. Follow-up complete / reasons described?** | **10. Addressed incomplete follow-up?** | **11.Appropriate analysis?** |
| --- | --- | --- | --- | --- | --- | --- | --- | --- | --- | --- | --- |
| Congyi Zheng | Yes | Yes | Yes | Yes | Yes | Yes | Yes | Yes | No / Partial | Unclear | Yes |
|  | CKM stages compared within the same national survey cohort; survey weighting reflects one population. | CKM staged by uniform definitions across the cohort (Methods/Results show stage-wise estimates). | Standard staging approach; survey procedures and SAS survey modules suggest uniform ascertainment. | Models adjusted for age, sex, urbanity, education, smoking, drinking, family history of CVD. | Adjusted Cox proportional hazards models. | Mortality modeled prospectively from baseline person-years across stages. | Cause of death verification referenced via China’s National Mortality Surveillance System. | Person-years and events by stage shown (Table 3); time-to-event analyses. | Authors report differences between those with and without follow-up (attrition exists). | Differences are described; specific corrective methods (e.g., IPW) not stated in visible text. | Cox regression using survey modules; adjusted HRs reported. |
| Min-Kuang Tsai | Yes | Yes | Yes | Yes | Yes | Yes | Unclear | Yes | Unclear | Unclear | Yes |
|  | All exposure groups (CKM stages) come from the same large health-screening cohort; single recruitment frame. | CKM staged within the same program using standardized procedures/instruments. | Explicitly states standardized screening with identical instruments. | Age, sex, education, smoking, alcohol, physical activity listed and used as covariates. | Multivariable Cox models adjusted for those covariates. | Baseline data from initial visit; mortality occurs after baseline (participants alive at entry). | Mortality ascertainment method (e.g., national registry linkage) is not explicit in the extracted text we have. (Methods likely contain it, but not visible in snippets.) | Median follow-up ≈16.5 years. | Cohort size and follow-up reported, but completeness/attrition handling not clearly described in visible text. | Not specified in the extracted text. | Cox models; proportional hazards checks and additional analyses reported. |
| Ruixin Zhu | Yes | Yes | Yes | Yes | Yes | Yes | Yes | Yes | Yes | Yes | Yes |
|  | Nationally representative NHANES cohort; complex sampling described. | CKM classified into 5 stages using defined criteria across cycles. | NHANES standard protocols; explicit CKM definition; survey weighting across 10 cycles. | Age, sex, race/ethnicity + education, marital, income, food security, insurance, employment, home ownership, access, alcohol, smoking, physical activity, cancer history. | Survey-weighted Cox models with staged adjustments. | Prospective follow-up for death after baseline; time origin defined at survey cycle. | Deaths identified by linkage to the National Death Index. | Median follow-up 8.3 years; events reported. | Passive NDI linkage; explicitly excluded 59 with missing death information. | Minimal loss via registry linkage; exclusions stated; sensitivity analyses (e.g., exclude first 2 years; competing risks). | Survey-weighted Cox; PH checked with Schoenfeld residuals. |
| Hongwei Ji | Yes | Yes | Yes | Yes | Yes | Yes | Yes | Yes | Unclear | Yes/Partial | Yes |
|  | Sex-stratified analyses within nationally representative NHANES; single sampling frame. | CKM stages defined using AHA criteria and applied uniformly. | Standard NHANES interviews/exams and biomarker assessments. | Adjusted for age, race, smoking, income, education; sex interaction tested. | Sex-specific multivariable Cox models; interaction terms; sensitivity with multiple imputation. | Prospective mortality linkage after baseline; follow-up to 2019. | Mortality via National Death Index linkage. | Median follow-up 13.3 years; 8745 deaths. | Large exclusions for missing data are reported; completeness of mortality linkage is implicit but not detailed in the excerpt. | Multiple imputation used for missing covariates; outcome linkage completeness not explicitly quantified in excerpt. | Survey-appropriate Cox models, PH checks, interaction tests; significance prespecified. |
| Mian Li | Yes | Yes | Yes | Yes | Yes | Yes | Yes | Yes | Yes | Yes | Yes |
|  | Multicentre community-based cohort; staging within one cohort. | CKM stages per AHA; LE8 metrics measured by standardized protocols/central lab. | Central laboratory assays; standardized OGTT and biomarker methods. | Models adjust for age, sex, education; additional mutual adjustment across LE8 metrics. | Fine-Gray competing-risk models with multivariable adjustment; extensive sensitivity analyses. | Incident CVD events adjudicated prospectively; baseline exclusions described. | Linkage to national disease surveillance, CVD registries, insurance; blinded adjudication. | Median 10.1 years; ~971k person-years; 7891 events. | Reports loss to follow-up counts and reasons (coverage gaps) and exclusions for incomplete outcomes. | Sensitivity analyses (exclude early events; subgrouping; multiple imputation for missing baseline/outcomes). | Fine-Gray models; spline analyses; PAF estimation; multivariable and sex/age stratified checks. |
| Ziyu Wang | Yes | Yes | Yes | Yes | Yes | Yes | Yes | Yes | Unclear | Unclear | Yes |
|  | All participants drawn from the CMCS, a multi-provincial Chinese community cohort with standardized protocols. | CKM health metrics and stages defined by AHA framework; applied uniformly across baseline survey. | Anthropometry, blood pressure, fasting labs, eGFR measured with validated methods; standardized questionnaires for lifestyle. | Adjusted for age, sex, smoking, drinking, education, snoring, physical activity, and diet. | Time-dependent Cox regression with multivariable adjustment, plus subgroup and sensitivity analyses. | Excluded participants with CVD at baseline. | Incident CVD events (CHD, stroke, plus HF/AF in subcohort) adjudicated by physicians and registry linkage. | Median 13.3 years (IQR 12.1–13.6), total >65,000 person-years. | Attrition described at re-examination (deaths, refusals, lost), but % follow-up completeness not fully quantified. | No detailed imputation; censoring applied. Sensitivity analyses excluded early deaths and medication users, but loss-to-follow-up handling not explicit. | Kaplan–Meier, Cox models, competing-risk regression, lag analyses, subgroup tests; statistically appropriate. |
| Haozhe Ding | Yes | Yes | Yes | Yes | Yes | Yes | Yes | Probably Yes | Unclear | Unclear | Yes |
|  | 9,116,728 adults from the same regional health check system (2019–2023). | CKM stages defined per AHA across the cohort. | Standard AHA staging; very large standardized dataset. | Age, sex, BMI, education, residence, occupation, marriage, smoking, drinking. | Multivariable Cox models specified. | Prospective follow-up of mortality after baseline checks. | Deaths from local death registrations. | Median follow-up 3.78 years reported. | Loss to follow-up not detailed in visible text. | Not described in visible sections. | Cox PH; Markov multi-state modeling; MI for missingness. |
| Na Li | Yes | Unclear | Unclear | Yes | Yes | Yes | Unclear | Yes | Unclear | Unclear | Yes |
|  | Single prospective cohort (Kailuan); exposure = CKM stage within cohort. | CKM staged, but explicit staging criteria not shown in visible text. | (As above) criteria not visible. | “Multivariable-adjusted” models reported. | Cox models with multivariable adjustment. | Prospective cohort; deaths accrue during follow-up. | Mortality ascertainment source not shown in visible text. | Median follow-up 15.0 (14.7–15.2) years. | Loss to follow-up not reported in visible sections. | Not described in visible sections. | Cox PH; dose-response trend; large N. |
| Kaitlin J. Mayne | Yes | Yes | Yes | Yes | Yes | Yes | Yes | Yes | Unclear | Unclear | Yes |
|  | All participants (40–69 y) recruited from 22 UK assessment centres in UK Biobank; same population base. | CKM stages defined by AHA framework; standardized baseline questionnaires, measurements, and lab samples. | Valid baseline measurements (e.g., BMI, BP, labs); CKM classification adapted for missing biomarkers but consistent with other large studies. | Models adjusted for age; interaction with sex tested and analyses stratified. | Stratified Cox regression and Kaplan–Meier absolute risk differences by sex. | Excluded prevalent CVD in classification; all participants alive at baseline. | Mortality from official NHS death certificates/registries. | Median 13.7 years (Q1–Q3: 13.0–14.5); 35,401 deaths, including 5406 CVD deaths. | UK Biobank generally has high linkage, but explicit % completeness or attrition not described in text. | No detailed description of handling loss to follow-up beyond censoring in survival models. | Cox regression, sensitivity analysis excluding early deaths, stratification by sex; appropriate statistical approach. |

## Figure S6. JBI Quality Appraisal Heatmap


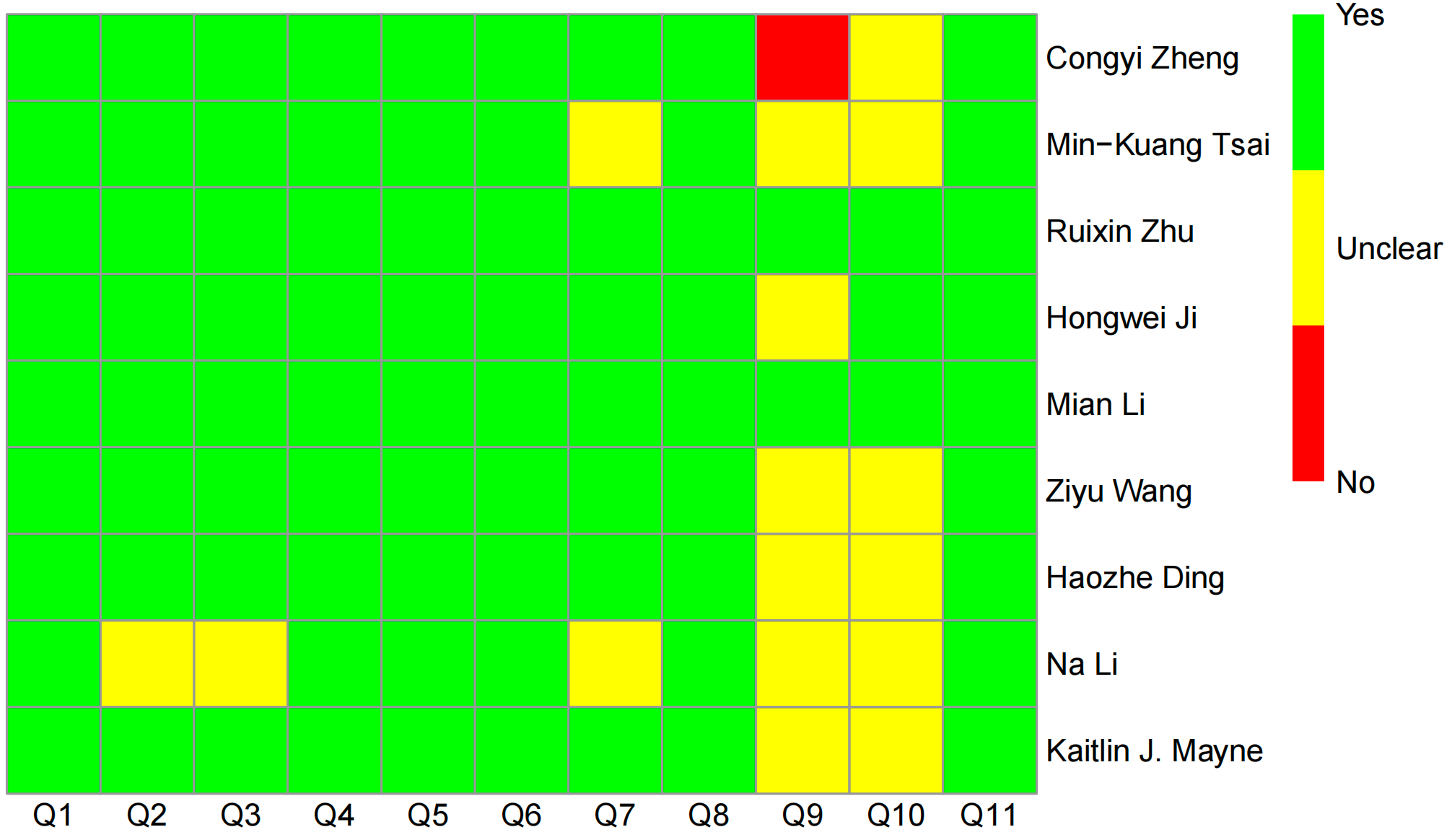

Supplement: Supplementary file 1 [file mmc1.docx]
